# Toward enhanced catalytic activity of magnetic nanoparticles integrated into 3D reduced graphene oxide for heterogeneous Fenton organic dye degradation

**DOI:** 10.1038/s41598-021-97712-7

**Published:** 2021-09-15

**Authors:** Fatemeh Sadegh, Nikolaos Politakos, Estibaliz Gonzalez de San Roman, Oihane Sanz, Ali Reza Modarresi-Alam, Radmila Tomovska

**Affiliations:** 1 POLYMAT, Facultad de Ciencias, Químicas, University of the Basque Country UPV/EHU, Joxe Mari Korta, Center - Avda. Tolosa, 72, 20018 San Sebastian, Spain; 2 Organic and Polymer Research Laboratory, Department of Chemistry, Faculty of Science, University of Sistan and Baluchestan, Zahedan, Iran; 3 Departamento de Química Aplicada, Facultad de Ciencias, Químicas, University of the Basque Country, UPV/EHU, P. Manuel de Lardizabal 3, 20018 San Sebastian, Spain; 4 Renewable Energies Research Institute, University of Sistan and Baluchestan, Zahedan, Iran; 5 Ikerbasque, The Basque Foundation for Science, Maria Diaz de Haro 3, 48013 Bilbao, Spain

**Keywords:** Chemical engineering, Environmental chemistry, Synthesis and processing

## Abstract

Composite Fenton nanocatalyst was prepared by water-based in situ creation of Fe_3_O_4_ nanoparticles integrated within the self-assembly 3D reduced graphene oxide (rGO) aerogel. The hybrid applied for the degradation of Acid Green 25 (AG-25) organic dye in an aqueous solution, in the presence of H_2_O_2_. By investigating the conditions that maximize the dye adsorption by the 3D composite, it was found that the pH of the solution should be adjusted between the pKa of the functional groups present on the rGO surface (carboxylic acid) and that of the dye (sulfonic acid) to promote electrostatic interactions dye—3D structure. Performed under these conditions, Fenton degradation of AG-25 in presence of H_2_O_2_ was completed in less than 30 min, including all the intermediate products, as demonstrated by MALDI–TOF–MS analysis of the aqueous solution after discoloration. Moreover, this was achieved in a solution with as high a dye concentration of 0.5 mg/mL, with only 10 mg of 3D composite catalyst, at room temperature and without additional energy input. The high performance was attributed to the creation of charge-transfer complex between rGO and Fe_3_O_4_ nanoparticles throughout covalent bond C–O–Fe, the formation of which was promoted by the in situ synthesis procedure. For the first time, up to the authors’ knowledge, AG-25 degradation mechanism was proposed.

## Introduction

Organic dyes are complex and toxic aromatic compounds, usually used to dye textiles or other products^1^. However, the effluents produced during dyeing processes are characterized by intense color and toxicity and have a strong impact on the environment. Therefore, the purification of these effluents to reuse the residual water or to release it in atmospheric waters is of huge importance, urged additionally by the universal lack of fresh water. One of the most investigated dye removal techniques is adsorption with solid adsorbents^2–4^ because the technique is low cost and simple^5,6^. Suitable solid adsorbents investigated for that aim are activated carbon, zeolite, clay, some metals, raw coal, wood chips, silica, metal oxides, and resin polymers^6–9^. The performance of these adsorbents was improved by decreasing their size to nanolevel, due to the increase in the active specific surface area^10–12^. Recently, graphene has been aroused as a good candidate for organic dye adsorption, due to its excellent and unique properties, such as high surface area, flexibility, and chemical, mechanical and thermal stability^13–16^. It was already demonstrated that graphene is an excellent adsorbent for various organic compounds, including organic dyes^17–21^. Moreover, the graphene based 3D aerogel nanostructures has shown to be even more efficient adsorbents^22–25^ that already have practical application for water purification. However, the adsorption of pollutants resolves only half of the problem, because the adsorbed dyes should be further either disposed or eliminated^26–28^. Simultaneous catalytic elimination of organic dyes from polluted water by high performance adsorbent, such as 3D rGO structures with anchored catalysts on the surface became promising for organic wastewaters treatment^29^. Between various catalytic processes studied, Fenton reaction over magnetic nanoparticles as catalysts combined with the 3D rGO aerogel structures has arisen as a potential advanced oxidation technique for organic dye elimination^30–34^.

Recently, we have proposed a green synthesis method for an efficient combination of 3D reduced graphene oxide (rGO) aerogel structure with magnetic iron nanoparticles, using aqueous media and mild synthesis conditions^35^. Few key issues of this work are considered behind the state-of-the-art: (1) Low energy synthesis process, achieved by the combination of simultaneous thermal and chemical reduction of GO and magnetic nanoparticles precursor; (2) Monolithic structure that allows easier implementation; (3) Use of MALDI–TOF MS techniques to analyze the degradation products. The process was evaluated using acid red organic azo dye, which was eliminated from the aqueous solution by the Fenton oxidation process in the presence of H_2_O_2_. The dye and the primary degradation products containing conjugated aromatic rings were completely degraded, however, some of the lower molar mass aromatic degradation products persisted after the treatment. The resulting nanocomposite catalyst has shown to be a solid base for efficient treatment technology development. Even though optimization of the Fenton process was performed based on the quantity of the solid adsorbent and the concentration of H_2_O_2_, further optimization is necessary to achieve complete mineralization of the adsorbed dye and all degradation products, which is a subject of the present work.

The oxidizing power of the Fenton system degradation mechanism is based on the creation ^•^OH radicals by the decomposition of H_2_O_2_ onto iron ion (Fe^2+^) promoter^36,37^. It was demonstrated previously that pH affects the iron ion oxidation, and subsequently it affects the rate of hydroxyl radicals generation^38^. The optimal pH range has been reported to be 2–4^39–42^. Increasing additionally the pH usually causes precipitation of iron in a hydroxide form. Nevertheless, the pH of the solution may further affect the quality, the rate and extent of the interactions between the catalyst and the dye. Considering that the heterogeneous catalysis consists of few elementary steps, pollutant adsorption, degradation reaction and products desorption, it is clear that degradation efficiency might be affected by the rate of each of these steps. So far, up to the authors’ best knowledge, the reported studies focused solely on the heterogeneous Fenton reaction degradation of various organic dyes^38–43^. However, if the adsorption process of the pollutant is inefficient and slow, it will negatively affect the degradation process. In other words, if the conditions are optimized for selective and fast pollutant adsorption, the efficiency of the overall process might be enhanced.

**Figure 1:**
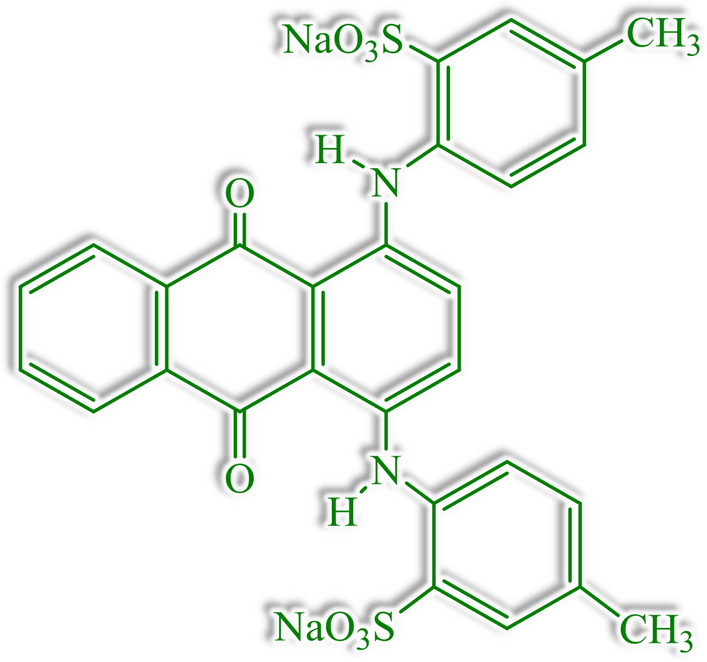
Chemical structure of AG-25 dyes.

In this work, by carefully studying solely the process of dye adsorption at different pHs, the optimal pH to maximize the interaction between the adsorbent and dye was determined, resulting in promoted adsorption, and subsequently, dye degradation in the presence of peroxide. Acid green azo organic dye (AG-25) was used as a model dye. It contains conjugated aromatic rings (Fig. 1), and, is far more complex than the acid red dye in our previous work^35^. It is worth mentioning that the process was performed under atmospheric conditions, without the use of the additional source of energy, and has presented 100% efficiency, degrading all the aromatic secondary degradation products, as demonstrated by MALDI–TOF–MS technique. To the best knowledge of the authors, this is the first study showing the optimization of adsorption step in heterogeneous Fenton process toward improved efficiency, as well as, proposed degradation mechanism for Fenton degradation of AG-25 organic dye.

## Experimental

### Materials

Ferrous sulfate (FeSO_4_·7H_2_O), poly(vinyl pyrrolidone) (PVP; 10,000 g mol^−1^), hydrogen peroxide (H_2_O_2_, 35 wt%), vitamin C or L-Ascorbic acid (AsA, ≥ 99.0%), ammonium hydroxide (NH_3_·H_2_O, 28–30 wt%) were supplied by Sigma-Aldrich. Graphene oxide (GO) aqueous dispersion with a concentration of 4 mg mL^−1^ (> 95% monolayer conten, pH 2.2–2.5).

Acid Green 25 (AG-25, C_28_H_22_N_2_NaO_8_S_2_) with molar mass 622.58 g mol^−1^ (Sigma-Aldrich) was used as the model pollutant dye. The structural formula of the dye is presented in Fig. 1. Deionized water (Milli-Q Millipore) was used throughout the experiments.

### Preparation of 3D-rGO/Fe_3_O_4_ nanostructures

The nanocatalysts were prepared by simultaneous reduction of GO and the precursor for the magnetic nanoparticles FeSO_4_·7H_2_O in colloidal aqueous dispersion at 90 °C. The process was performed either thermally or by combined thermal and chemical reduction using AsA as a reducing agent.

An aqueous dispersion of 4 mg mL^−1^ GO was ultrasonicated (Hielscher Sonicator-UIS250v) with an amplitude of 70% and energy pulsed at 0.5 Hz for 1 h at room temperature (RT). Then, AsA was added to this dispersion (AsA : GO = 1 : 2) and it was stirred for 30 min at room temperature (RT). This step was avoided when solely thermal reduction was used to produce the nanostructures. Afterward, 0.2 M FeSO_4_·7H_2_O was added (at a molar ratio of rGO : FeSO_4_ = 1 : 2) and the pH was adjusted to 11 by dropwise addition of NH_3_·H_2_O. Excess of FeSO_4_ was used to achieve high nanoparticle loading. The obtained dispersion was transferred into an oven and kept at 90 °C for 2 h without stirring. As a result, monolithic 3D composite structures rGO/Fe_3_O_4_ were obtained. Often, instead of one monolith, a few smaller monolithic pieces were formed. To produce neat magnetic nanoparticles, the same procedure was followed, without the addition of GO to the aqueous dispersion. The produced nanostructures were washed with DI water and frozen under liquid nitrogen, and subsequently freeze-dried (lyophilized) for 3 days to completely remove the water, producing the 3D aerogel structures.

**Figure 2:**
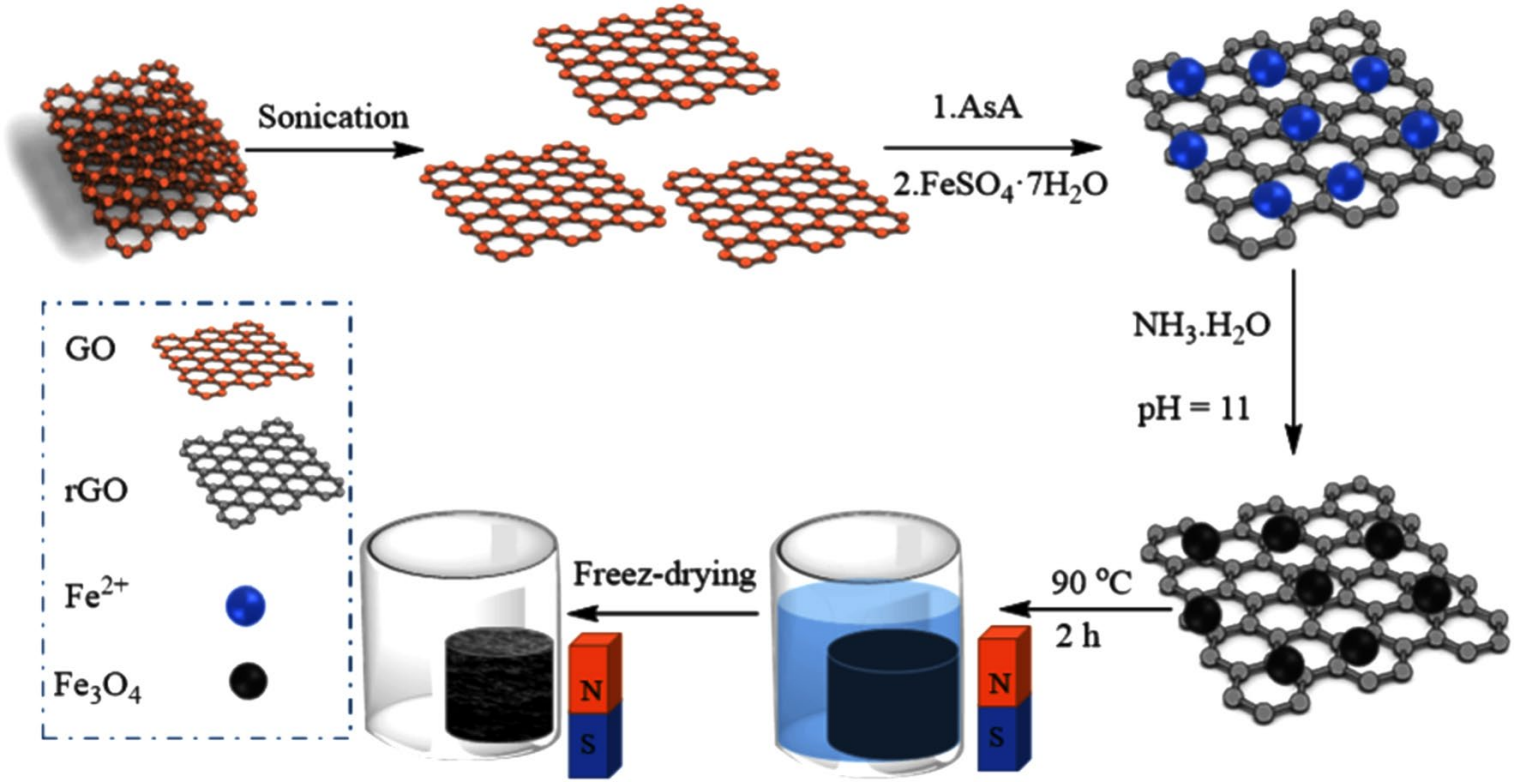
Schematic diagram of the preparation of the 3D-rGO/Fe_3_O_4_ nanostructures.

For comparison aim, the composite sample produced by reduction with AsA was dried as well conventionally in an oven at 50 °C for 8 h. The preparation process is schematically presented in Fig. 2. In one experiment, before the reduction process, PVP as a shape controlling agent was added to investigate its effect on the porous structure and morphology. The reaction conditions are summarized in Table 1.

**Table 1:** Nomenclature of 3D materials and the conditions for their synthesis.

Nanocalatysts nomenclature	GO: Fe_3_O_4_ wt ratio	GO:AsA wt ratio	PVP %	The drying process
Neat 3D-rGO	–	1:2	–	Lyophilization
Th-rGO/Fe_3_O_4_/PVP	1:2	no AsA	3%	Lyophilization
Ch-rGO/Fe_3_O_4_	1:2	1:2	–	Lyophilization and 50 °C, 8 h
Th-rGO/Fe_3_O_4_	1:2	no AsA	–	Lyophilization

### Characterization

The 3D-rGO/Fe_3_O_4_ nanocomposites were characterized by thermogravimetric analysis (TGA, TA Instruments Q500), by heating from 25 to 800 °C at a rate of 10 °C min^−1^ under nitrogen. The morphology of the nanocomposites was evaluated using a Hitachi TM3030 tabletop Scanning Electron Microscope (SEM), equipped with an energy dispersive X-ray (EDX) instrument at an accelerating voltage of 15 kV. The textural properties of the materials were studied by N_2_ adsorption isotherms measured at − 196 °C with an ASAP 2020 instrument from Micromeritics. Before the analysis, the samples were degassed at 120 °C for 5 h. The surface area (SBET) was determined by the BET (Brunauer–Emmett–Teller) method. The total pore volume (Vp) was determined from the amount of nitrogen adsorbed at P/P0 = 0.95. The pore size distribution was analyzed by the BJH (Barrett–Joyner–Halenda) method from the adsorption branch of the isotherm and the average pore diameter is obtained as Dpore = 4V/SBET.

The crystalline phases were studied by X-ray diffraction (XRD) operating at a scanning rate of 10°/min from 10° to 80° (Philips PW1730) using Cu K*α* radiation. The magnetic properties of the nanoparticles have been examined using a vibrating sample magnetometer (VSM, AGFM/VSM 3886).

To determine the surface chemistry of the 3D structures, XPS analyses were performed on a SPECS Sage HR 100 spectrometer with a non-monochromatic X-ray source (aluminium Ka line of 1486.7 eV energy and 252 W) placed perpendiculary to the analyzer axis and calibrated using the 3d5/2 line of Ag with a full width at half maximum (FWHM) of 1.1 eV. The selected spectra resolution was 15 eV pass energy and 0.15 eV per step. The measurements were performed at a pressure around 8 × 10^–8^ mbar in an ultra-high vacuum chamber. For the Fe 2p and Fe 3p regions, the pass energy was 10 eV with 15 acquisitions.

TEM analysis was carried out with the TECNAI G2 20 TWIN, equipped with LaB6 filament. The 3D sample was crushed in a mortar and the obtained powder was dispersed in ethanol, which was drop cast over the grid.

### Removal of AG-25 from aqueous solution

With the composite 3D structures placed in AG-25 aqueous solution, two types of experiments were performed: simple dye adsorption and dye degradation. For both of these experiments, 3D nanostructure (0.010 and 0.020 g) was placed in aqueous solutions of AG-25 with different dye concentrations (0.01–0.5 mg mL^−1^). For the dye degradation experiment, H_2_O_2_ was added (2 mL of 0.01–0.05 M) to an aqueous solution containing already the 3D structure. The discoloration experiments were performed at RT at different pH, changed in the range of 3–10. Samples of the aqueous solution were extracted at certain time intervals (5–180 min) and were analyzed by UV/Vis spectrophotometer at a wavelength of 608 nm to determine the actual dye concentration.

After each experiment, the 3D-rGO/Fe_3_O_4_ adsorbent was separated by an external magnet and the final dye concentration was determined. The removal efficiency (R%) of AG-25 by the nanocatalysts was calculated using Eq. (1)^44^

1 $$ \% {\text{ R}} = \frac{{{\text{C}}_{{\text{o}}} - {\text{C}}_{{\text{e}}} }}{{{\text{C}}_{{\text{o}}} }} \times 100 $$

where C_o_ and C_e_ are the liquid phase initial and equilibrium concentrations of the AG-25 dye, respectively.

To demonstrate the dye degradation, MALDI–TOF–MS measurements were performed on a Bruker Autoflex Speed system (Bruker, Germany) equipped with a Smartbeam-II laser (Nd:YAG, 355 nm, 2 kHz). Spectra were acquired in reflectron mode; each mass spectrum was the average of 10,000 shots. The laser power was adjusted during the experiments. Each sample was characterized as received after Fenton’s reaction with AG-25 without using a matrix. Approximately 0.5 μL of the obtained solution was spotted by hand on the ground steel target plate and allowed to dry in the air.

Spectra were accumulated and processed using FlexControl (v3.4) and FlexAnalysis software (v3.4), respectively. Peaks were detected in SNAP mode with a signal-to-noise threshold of 3.00 before being processed with a Savitzky–Golay smoothing algorithm (0.05 m/z width, one cycle) and “TopHat” baseline subtraction. External calibration was performed in quadratic mode with a mixture of different polystyrene standards (PS, Varian).

## Results and discussion

**Figure 3:**
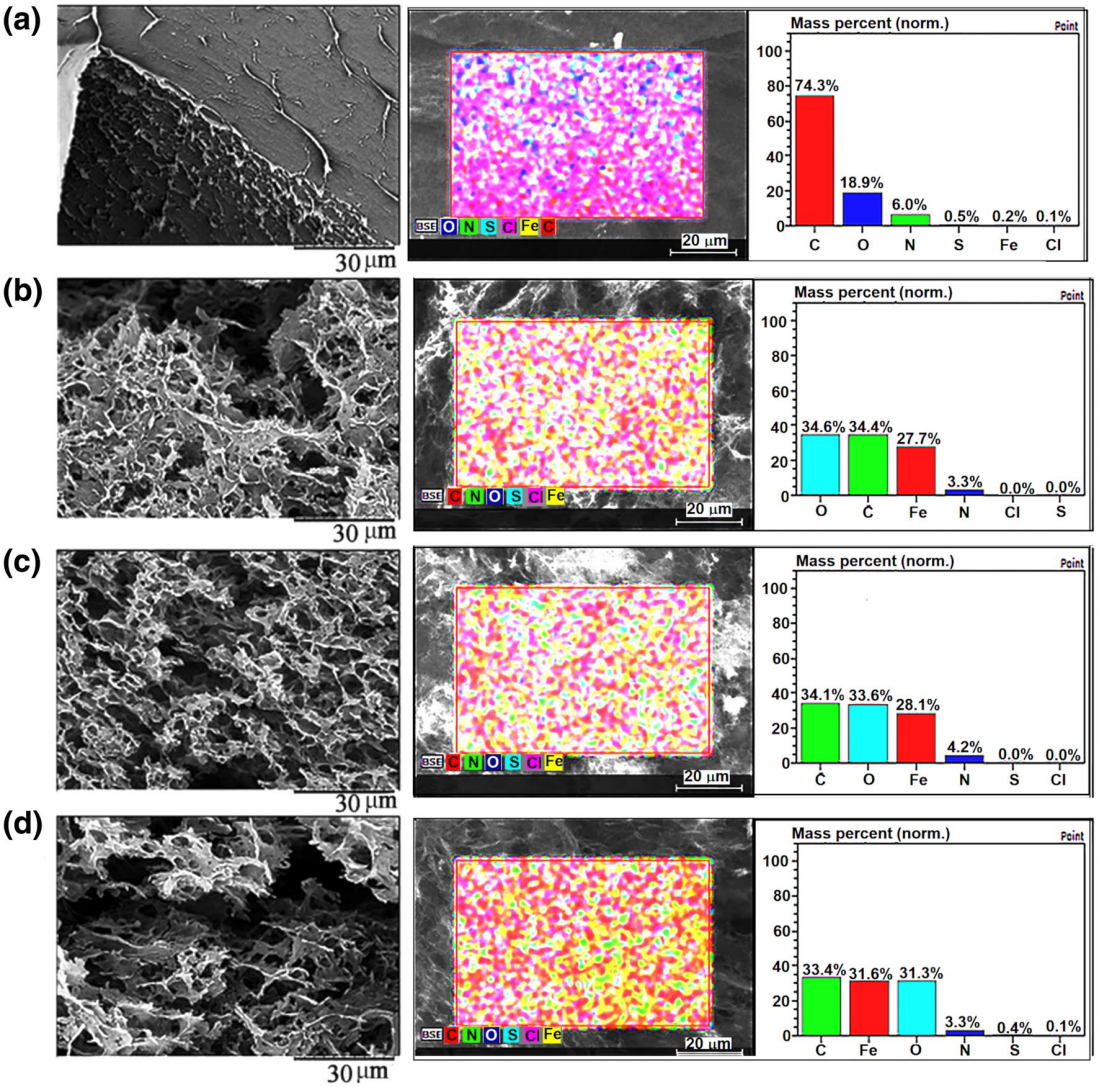
Surface morphology, mapping and elemental composition of the 3D nanocomposites (**a**) Neat 3D-rGO; (**b**) Th-rGO/Fe_3_O_4_/PVP; (**c**) Ch-rGO/Fe_3_O_4_; and (**d**) Th-rGO/Fe_3_O_4_.

Under all investigated synthesis conditions shown in Table 1, 3D materials are produced. The 3D structure was formed due to the self-assembly of the GO platelets, which during reduction turned to be highly hydrophobic. Hence, they joined together to decrease the interfacial tension raised in the system due to chemical changes on the rGO^45^. The magnetic nanoparticles formed in situ were spontaneously incorporated into the structure. The magnetic nanoparticles nucleated onto the GO nanoplatelets and grow as the reduction progressed on the nanoplatelets’ surface, as was observed previously^35^. In Fig. 3, the morphology determined by SEM of the 3D structures obtained by freeze-drying of the monoliths and their chemical composition determined by EDX are presented, including the EDX mapping.

The SEM images in Fig. 3a–d present porous 3D morphology of the nanocomposites. The neat 3D-rGO is a rather compact 3D material at the scale shown in Fig. 3a. The presence of magnetic nanoparticles affected the morphology, which became more porous, presenting the increasing number of micrometer pores, as it is shown in Fig. 3b–d. A similar effect was noticed in our previous work, which was assigned to the fact that magnetic nanoparticles attached to the rGO surface actuated as spacers and prevented complete re-aggregation of rGO platelets during the self-assembly^35^. Therefore, the formed composite nanostructures are fluffier than the neat 3D-rGO. The elemental distribution mapping in Fig. 3b–d demonstrates the presence of magnetic nanoparticles in the nanocomposites (Fe element is denoted in red), whereas in Fig. 3a, one can observe the carbon reach surface (carbon is denoted in red in this image). The elemental compositions additionally demonstrated this evidence. Therefore, in neat-rGO nanostructure, the material is composed roughly of about 74% carbon and 19% oxygen, with small nitrogen content probably coming from the ammonia used to adjust pH. In the composite nanostructures, the relative quantities of carbon dropped importantly, causing increase the Fe amount. However, the elemental composition between the nanocomposites is rather similar, which means that the employed conditions did not affect it. Moreover, in the EDX maps, it can be observed that the magnetic nanoparticles are uniformly distributed all over the rGO matrix.

It is worth noting that the 3D hydrogel structure was freeze-dried because in the case of conventional heating, a significant decrease in the volume of tCh-rGO/Fe_3_O_4_ was observed, as it is shown in Fig. S1. The oven-drying process had a notable effect on the morphology, too, as can be seen in Fig. S1, flower morphology was obtained in this case, made of highly wrinkled rGO nanoplatelets. This material was not further investigated, due to the rigid nature and difficult handling, as the 3D structures dried conventionally were not stable, and we show them because of the peculiar morphology, which can suggests different types of application.

**Figure 4:**
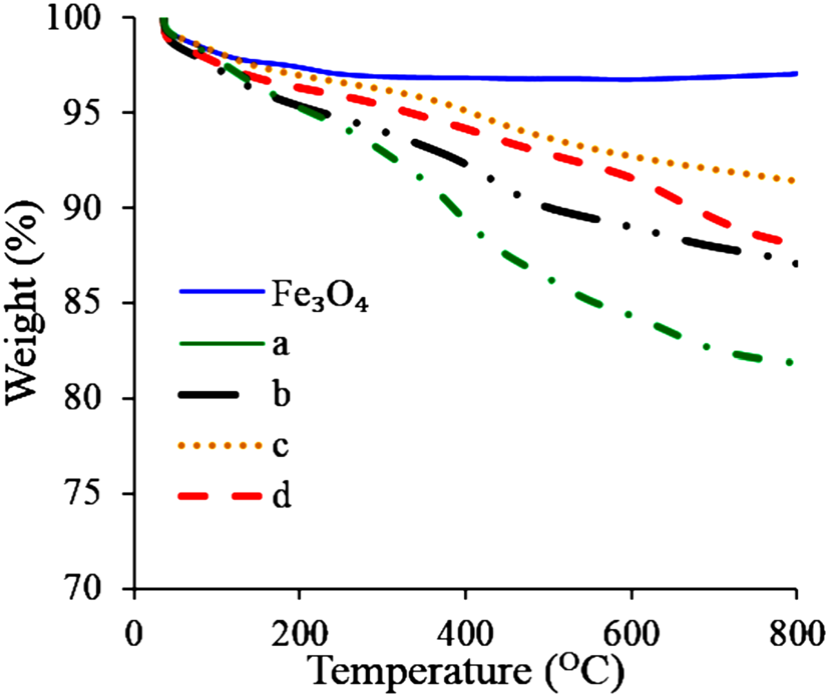
TGA results of neat Fe_3_O_4_ nanoparticles; (**a**) neat3D-rGO; (**b**) Th-rGO/Fe_3_O_4_/PVP; (**c**) Ch-rGO/Fe_3_O_4_; and (**d**) Th-rGO/Fe_3_O_4_.

The 3D structures were analyzed by TGA to determine the thermal stability of the nanocomposites and to obtain additional information on the structure and interaction within the composites. Figure 4 shows the weight loss curves obtained by TGA of neat Fe_3_O_4_ nanoparticles, the neat 3D-rGO and nanocomposite materials. The TGA study shows that the nanocomposite structures present thermal stability and thermal degradation behavior between the neat components rGO and magnetic nanoparticles. The synthesis conditions influence the thermal stability.

The most thermally stable composite is Ch-rGO/Fe_3_O_4_, obtained by simultaneous chemical and thermal reduction. The synthesis performed by both AsA and temperature is likely the reason for the improved thermal stability because it provides conditions for a faster reduction process, resulting in a sudden confluence of the nanoplatelets and substantially more compact structure formation. Consequently, the more compact structure needed more energy to be thermally degraded. The thermally weakest structure was the one obtained in the presence of PVP, which is a colloidal stabilizer. When adsorbed on the surface of rGO platelets, PVP decreases the interface tension between rGO and water, which is likely the driving force for the nanoplatelet confluence. Therefore, the resulting structure is less compact and easier to be thermally degraded.

The specific surface area of the nanocomposite is an important property that influences the adsorption and catalytic capability. It depends on the textural properties of the nanocomposites, which were determined from the N_2_ adsorption–desorption isotherms, presented in Fig. S2.

The isotherms shown in Fig. S2 are of type IV (IUPAC classification^46^), indicating that the nanostructures are mesoporous. The existence of a hysteresis loop between adsorption–desorption process accounts for irregular pore shapes in which the capillary condensation phenomenon occurred^47^. The textural properties of the nanocomposites are presented in Table 2. They correspond to the indication obtained from TGA analysis. The highest BET surface area was obtained for neat 3D-rGO and Ch-rGO/Fe_3_O_4_, both of them obtained from combined chemical (AsA) and thermal reduction. The addition of magnetic nanoparticles negatively influenced the porous structure, as the average diameter of the pores increased, likely because the nanoparticles strongly adsorbed on the rGO nanoplatelets, acting as a spacer in the moment of nanoplatelets stacking, but as well making the nanoplatelts more rigid. Consequently, they formed larger pores between 10 and 100 nm (larger distance between the nanoplatelets) (Fig. S2, inset). If we compare the neat 3D-rGO and Ch-rGO/Fe_3_O_4_, which were obtained exactly under the same conditions, the presence of Fe_3_O_4_ nanoparticles affected positively the porosity, as higher pore volume was obtained, resulting in the largest BET surface area obtained in this work of about 266 m^2^ g^−1^ (Table 2). In addition, this preparation method allows maintaining the pores of the 3D-rGO. This can be result on a less rGO platelets aggregation in the presence of magnetic nanoparticles, thus the walls of the pores are thicker in the neat rGO structure, which decreases its specific surface area. SEM image (Fig. 3a) demonstrates this additionally.

**Table 2:** Textural properties of the nanostructures.

Nanocalatysts	Surface área (m^2^ g^−1^)	Pore volume (cm^3^ g^−1^)	Average pore size (nm)
3D-rGO	258	0.22	3
Th-rGO/Fe_3_O_4_/PVP	164	0.33	8
Ch-rGO/Fe_3_O_4_	266	0.40	5
Th-rGO/Fe_3_O_4_	204	0.35	6

When the nanostructures were synthesized solely by thermal reduction, in both cases the composite structures are more porous than the neat 3D-rGO. Nevertheless, the porosity decreased with respect to Ch-rGO/Fe_3_O_4_, resulting in a lower BET of 204 m^2^ g^−1^ for Th-rGO/Fe_3_O_4_ and 164 m^2^ g^−1^ for Th-rGO/Fe_3_O_4_/PVP, due to an important decrement of 1–10 nm of the pore diameter (Fig. S2, inset). The presence of PVP in such cases, furthermore affected negatively the textural properties.

Based on the thorough characterization of the developed composites, Ch-rGO/Fe_3_O_4_ was selected for the study of the discoloration process of AG-25 aqueous solution. Before the discoloration study, the chemical composition of Ch-rGO/Fe_3_O_4_ was examined by XPS to determine the rGO–Fe bonding, since it may be decisive for catalytic activity.

**Figure 5:**
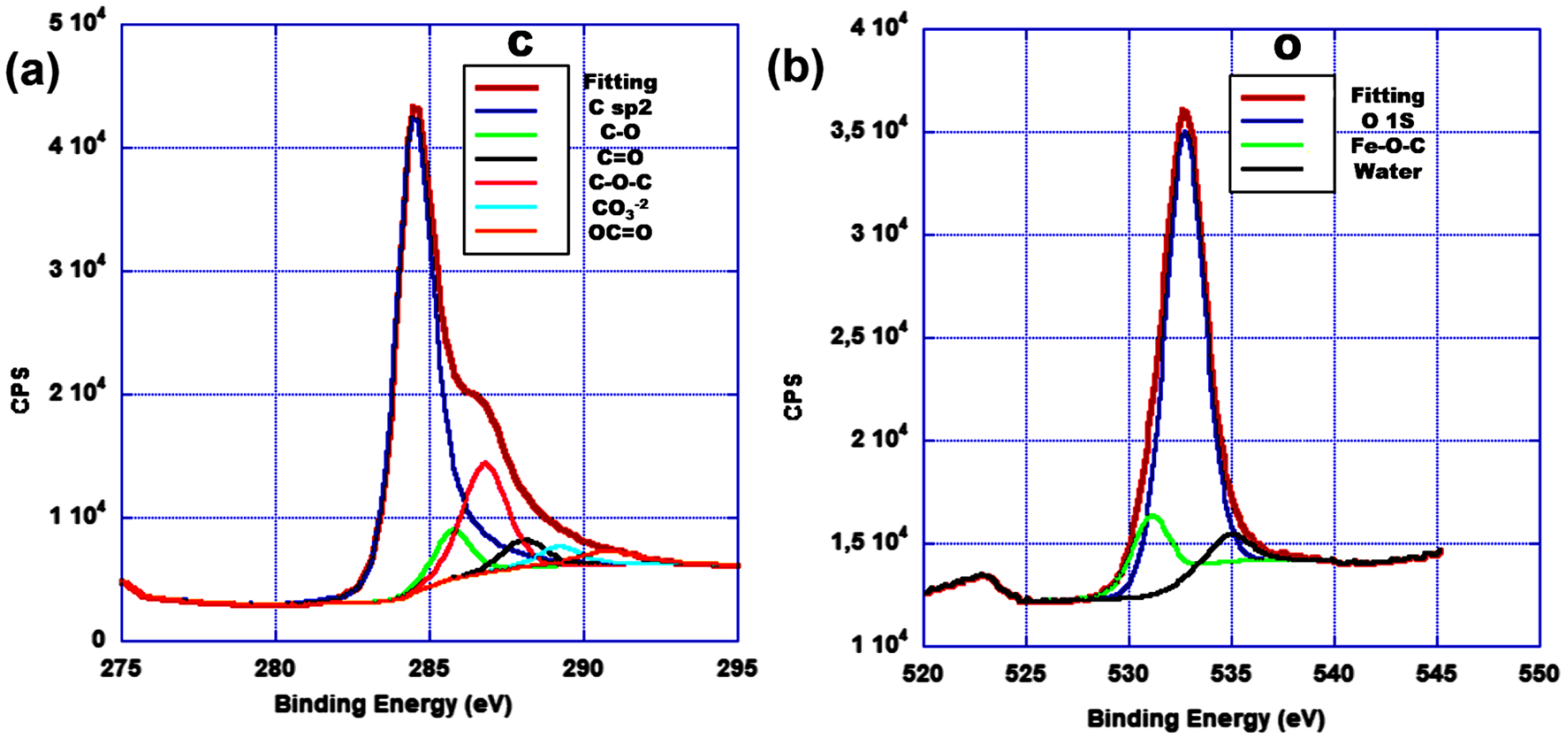
XPS spectra of Ch-rGO/Fe_3_O_4_ (**a**) C 1s and (**b**) O 1s core-level spectra.

In Fig. 5a, the C1s region after deconvolution shows that the main contribution originates from sp^2^ C, the peak centered at 284.6 eV and lesser quantity of C–O–C at 286.1 eV, followed by few contributions of C–O, C=O, CO_3_^–2^ and O–C=O. No evidence of Fe–C bonding was observed. The wide peak observed in Fig. 5b denoting O 1s region displays the major presence of C–O, some oxygen from humidity and a peak observed at 530.9 eV, assigned to the Fe–O–C bond. The presence of this peak demonstrates that the magnetic nanoparticles were strongly bonded to the 3D rGO structure by a covalent bond established between graphene and magnetic nanoparticles through oxygen. This bond has already been observed in similar structures^35,48^, and was promoted by the in situ creation of the Fe_3_O_4_ NP. The proposed routes toward establishing this structure are based on either replacement of hydrogen atoms in the carboxylic group by bidentate coordination with an iron atom, or iron catalyzed ring opening reaction of epoxy groups^48^.

The XRD patterns of rGO and Ch-rGO/Fe_3_O_4_ were presented in Fig. S3. As shown in Fig. S3a, a broad peak appeared at about 25.81° corresponds to the (002) plane of rGO. The diffraction peaks observed in Fig. S3b, centered at 2θ of 29.34, 35.71, 36.25, 43.82, 53.87, 57.40, and 63.13° can be assigned to (220), (311), (222), (400), (422), (511), and (440) reflections, respectively, for Fe_3_O_4_ in Ch-rGO/Fe_3_O_4_ nanostructure (JCPDS No. 72-2303)^49,50^.

The magnetization hysteresis loops of curves of Fe_3_O_4_ and Ch-rGO/Fe_3_O_4_ were measured by VSM at room temperature. From Fig. S4, it is evident that the saturation magnetization values for Fe_3_O_4_ and Ch-rGO/Fe_3_O_4_ nanocatalyst were ~ 58/20 and 24 emu g^−1^, respectively, which demonstrate the magnetic properties of the nanoparticles distributed within 3D rGO structure^51^.

**Figure 6:**
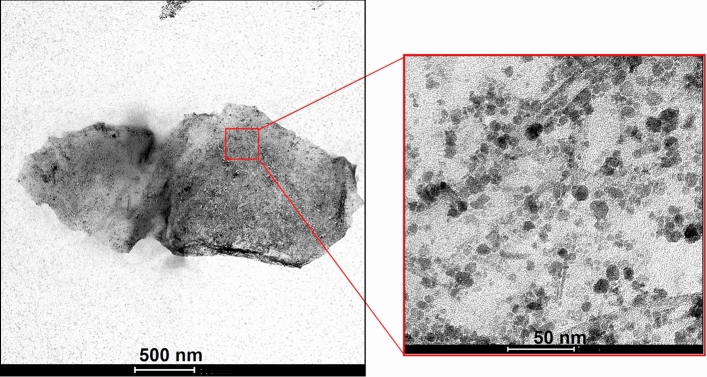
TEM images of Ch-rGO/Fe_3_O_4_.

The distribution and size of the magnetic nanoparticles in Ch-rGO/Fe_3_O_4_ were studied by TEM. Figure 6 presents the representative TEM images, where a single rGO/Fe_3_O_4_ hybrid nanoplatelet is shown in Fig. 6a, whereas in Fig. 6b, a magnified area of the same platelet is shown. The rGO platelet is covered completely by magnetic nanoparticles. The size distribution of the nanoparticles is within a range of 5–20 nm.

### Discoloration of AG-25 aqueous solutions by adsorption

The 3D structured monolith Ch-rGO/Fe_3_O_4_ was used as an adsorbent and nanocatalyst for AG-25 azo dye elimination from model polluted aqueous solutions. The effect of dye concentration, pH, and the adsorbent dose was studied to find out the optimal conditions. In the first part of the study of the discoloration ability of the Ch-rGO/Fe_3_O_4_ 3D material, the investigation was focused on the adsorption of the AG-25 azo organic dye. It was compared with neat 3D-rGO. Figure S5 shows a schematic illustration of the adsorption process for the AG-25 using Ch-rGO/Fe_3_O_4_.

**Figure 7:**
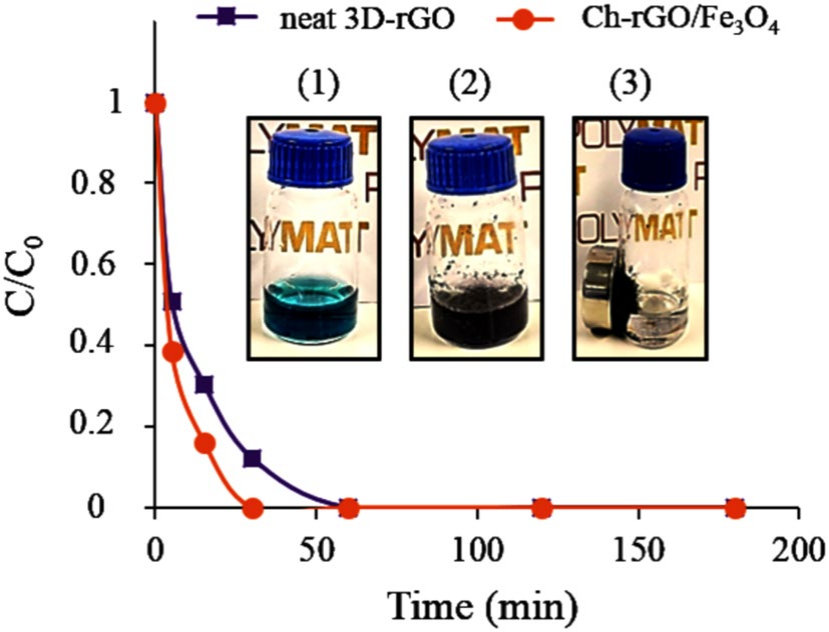
The effect of adsorbent kind on adsorption of AG-25 by neat rGO and Ch-rGO/Fe_3_O_4_. Experimental conditions: adsorbent dosage 0.010 g, dye concentration 0.01 mg mL^−1^, RT, and natural pH; Inset: Photos of the discoloration process and separation of the nanostructure afterward.

According to Fig. S5, 0.010 g of Ch-rGO/Fe_3_O_4_ or 3D-rGO nanostructure was placed in the AG-25 aqueous solution with a dye concentration of 0.5 mg mL^−1^ without adjusting the pH. As it is shown in Fig. 7, for composite nanostructure Ch-rGO/Fe_3_O_4_ complete discoloration occurred in 30 min, whereas neat 3D-rGO nanostructure needed 60 min to discolor completely the solution. The enhanced performance of the composite nanostructure is due to the increased porosity and the contribution of very small pores to the larger specific surface area offered by the Ch-rGO/Fe_3_O_4_ for the capture of dye molecules. The inset in Fig. 7 shows the photos of the discoloration process: (1) presents the initial dye solution; (2) presents the same solution after the addition of nanocomposite catalyst; and (3) presents the effective and easy separation of the nanostructure from the aqueous solution after complete discoloration by an external magnet.

**Figure 8:**
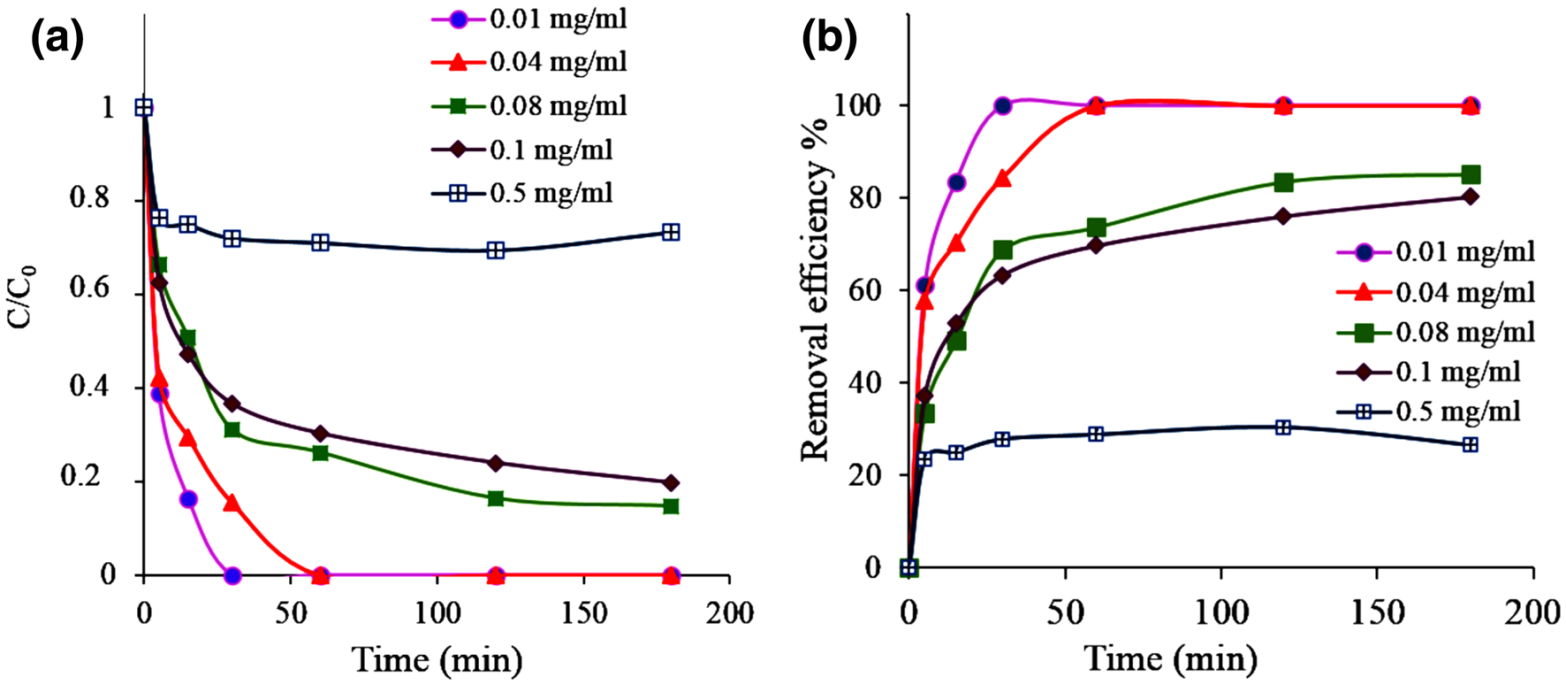
The effect of initial dye concentration on adsorption of AG-25 by Ch-rGO/Fe_3_O_4_. Experimental conditions: adsorbent dose 0.010 g, RT, and natural pH.

Figure 8 presents the kinetic curves and the process efficiency as a function of the dye concentration, studied in the range of 0.01–0.5 mg mL^−1^ at a constant amount of composite nanostructure Ch-rGO/Fe_3_O_4_ (0.010 g). The adsorption rate dropped importantly at higher dye concentration (Fig. 8a), as well as the removal efficiency (Fig. 8b). With the studied amount of nanostructure, the maximum concentration of the dye until which the process is efficient (100% removal rate) is 0.04 mg mL^−1^. In the case of a high dye concentration as 0.5 mg mL^−1^, the process efficiency is about 26%. The performance decreased likely because of the saturation of the adsorption sites with the dye molecules as its concentration increased. The adsorption rate initially is very high and similar for all dye concentrations, however, as the surface of the adsorbents became saturated, the rate dropped substantially. This drop is larger for a larger dye concentration. Afterward, the kinetic curves present saturation of the adsorbent and no further dye adsorption. This indicates that the process should be optimized further.

It is well known that the Fenton reaction, for maximum performance, should be performed at pH in a range of 3–4 because at higher pH the iron precipitates in a form of hydroxide^39–42^. This means that there is a loss of valuable ferrous ions and H_2_O_2_, turning the reaction inefficient. However, this is so if homogeneous Fenton reactions are considered. In the case of the heterogeneous process, the pH of the solution additionally affects the interactions adsorbent–dye because the pH determines the adsorbent surface charge, degree of ionization of pollutant, separation of functional groups on the active sites of the adsorbent as well as the structure of the dye molecule. If the interaction conditions are unfavorable, there will be less or no contact between the dye molecule and the magnetic nanoparticle catalysts. Therefore, it would be of great importance to study these interactions conditions at different pH. To distinguish them from the Fenton reaction effects, the influence of pH was studied in the absence of H_2_O_2_.

**Figure 9:**
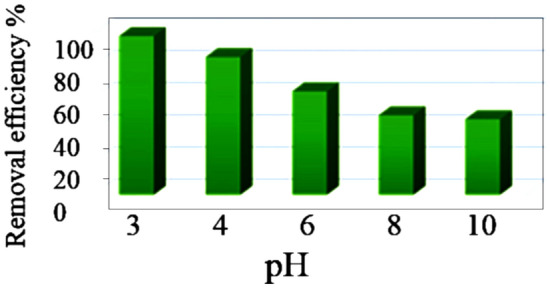
The effect of pH on the adsorption of AG-25 by Ch-rGO/Fe_3_O_4_. Experimental conditions: adsorbent contact time 180 min, dye concentration 0.1 mg mL^−1^, RT, and adsorbent dose 0.010 g.

In the case of 0.1 mg mL^−1^ dye concentration, the maximum-achieved adsorption efficiency using 0.010 g adsorbent was close to 80%. This efficiency was obtained at the natural pH of the dispersion without any adjustment. The adsorption process was then studied in pH range of 3–10. Figure 9 shows the effect of pH on the adsorption efficiency of AG-25 by Ch-rGO/Fe_3_O_4_.

pH augmentation from 3 to 10 adversely affects the adsorption process, thus, the AG-25 adsorption efficiency dropped from about 98% at pH = 3 to as low as 40% at pH = 10. Considering that there are some carboxylic functionalities on the surface of the rGO based structure, at pH = 3, which is lower than pKa = 4 of carboxylic acids^52^, most of them will be in protonated form. However, in such conditions, the sulfonic acid groups in AG-25 are in anionic form, as their pKa is 2.8^53^, which promotes ion–dipole interactions between sulfonic anion and the carboxylic group. Likely, by increasing the pH, carboxylic groups are turned into deprotonated (anionic) form and electrostatic repulsion occur between the graphenic carboxylic anion and the anionic sulfonic acid groups in the dye, resulting in the drop of the adsorption amounts, as shown in Fig. 9. In such cases, only the interactions of the neat graphenic areas on the 3D structures with the aromatic conjugated structure of AG-25 dye likely build π–π interactions with graphene. This results in a removal efficiency in a range of 60–40%, and likely will affect Fenton reaction, making it slower and less efficient.

For the specific chemistry of the selected organic dye AG-25 and rGO structure, the pH of 3 has shown maximum removal efficiency. This result would be probably be affected if another type of chemistry for adsorbent and dye were selected. However, it seems that the best performance pH for the adsorbent–dye interactions cross the threshold of the suggested pH range for the best performance of magnetic nanoparticles as Fenton catalyst.

**Figure 10:**
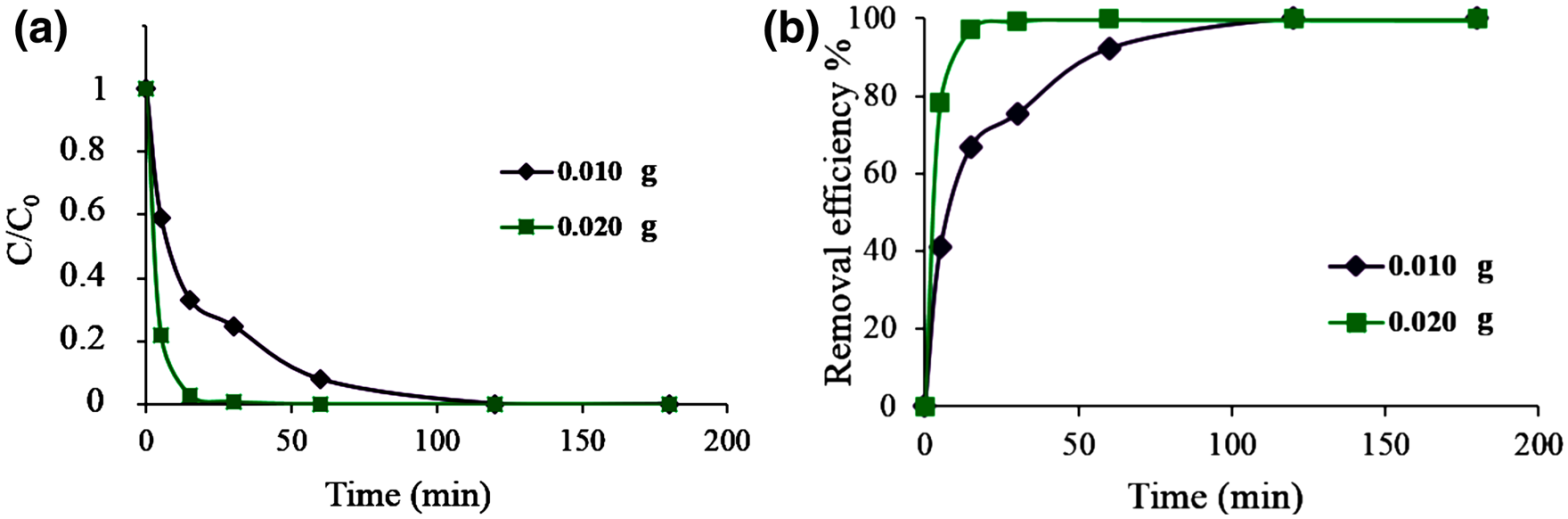
The effect of adsorbent dosage on the adsorption of AG-25 by Ch-rGO/Fe_3_O_4_. Experimental conditions: dye concentration 0.1 mg mL^−1^, RT, and pH = 3.

Furthermore, the effect of the adsorbent dose on adsorption capacity and the removal efficiency was studied, at pH = 3. For that aim, the amount of the adsorbent used (0.010 g) was duplicated (0.02 g). The kinetic curves and the removal efficiency are compared in Fig. 10a,b.

Figure 10 showed that the solution of AG-25 dye with a concentration of 0.1 mg mL^−1^ was discolored in 2 h by 0.010 g adsorbent. However, when a double amount of adsorbent was used, the removal time was decreased to less than 30 min, for which the efficiency is 100%. It is worth mentioning that the removal efficiency of this reaction was noticeably augmented, as it is similar to the removal efficiency presented in Fig. 8 obtained for one order of magnitude more diluted dye solutions (0.01 mg mL^−1^). The last result is due to the doubled amount of adsorbent that offers much more adsorption sites and due to the pH of 3 that promoted dye–adsorbent interactions.

### Mechanism of the AG-25 adsorption by 3D rGO/Fe_3_O_4_ composite nanostructure

The proposed adsorption mechanism is presented in Fig. S6. The aromatic conjugated structure of AG-25 dye provides the opportunity of building strong π–π interactions with graphene. Neat graphenic areas in the studied 3D materials provide a base for establishing these interactions. However, the presence of functional groups on the graphene surface, the ionic state of which can be tailored by the pH of the solution, provides further adsorption sites, by establishing ionic interactions with the dye molecule. An acidic pH of 3 was found to have to determine the effect on dye adsorption. The pH should be adjusted to be in-between that of pKa of graphene carboxylic groups and pKa of sulfonic acid group of AG-25 to maximize the adsorption capacity of the 3D structure.

### Degradation of AG-25 by Fenton reaction

**Figure 11:**
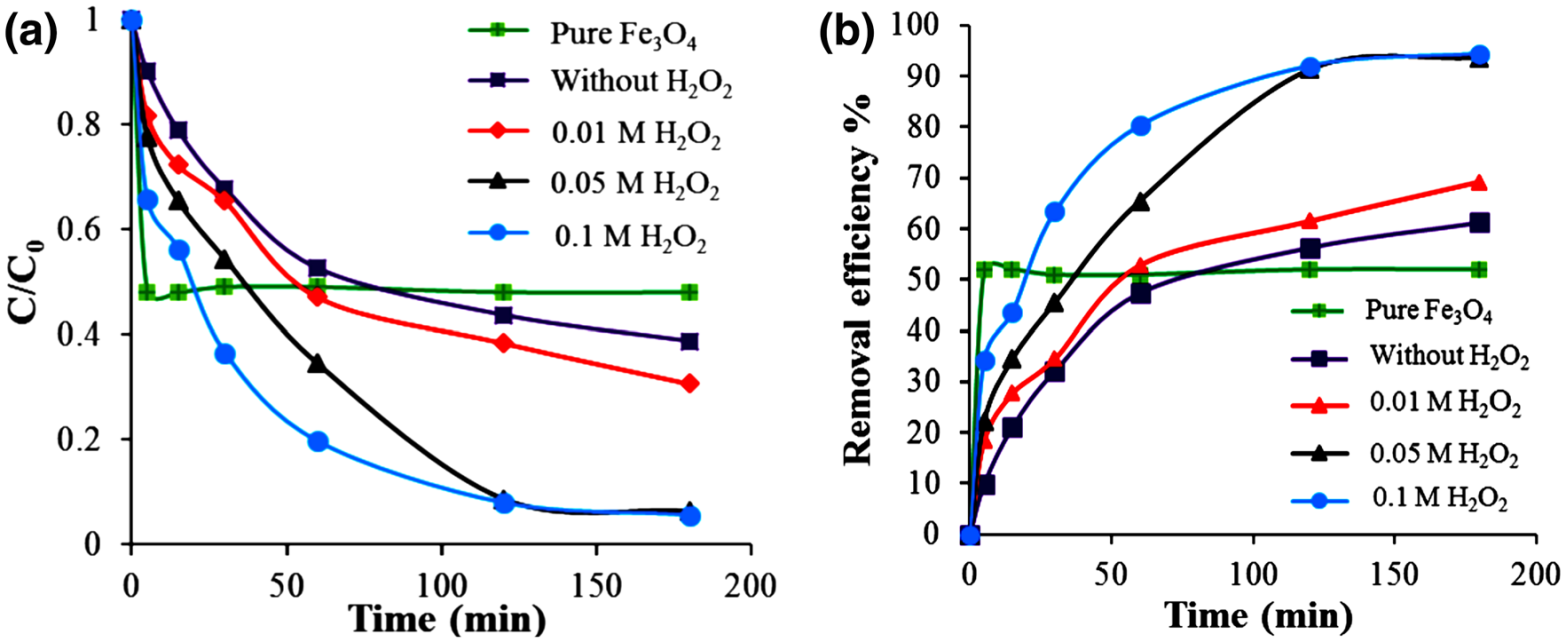
The effect of H_2_O_2_ dosage on the adsorption/degradation of AG-25 by Ch-rGO/Fe_3_O_4_. The efficiency of neat magnetic nanoparticles on AG-25 degradation for 0.1 M H_2_O_2_. Experimental conditions: dye concentration 0.5 mg mL^−1^, RT, 0.010 g adsorbent and pH = 3.

When the aqueous solutions of AG-25 were exposed to H_2_O_2_ in the presence of the Ch-rGO/Fe_3_O_4_ as a Fenton catalyst, the peroxide creates hydroxyl radicals on the surface of the catalysts for oxidation of the pollutant^35,54^. The effect of H_2_O_2_ concentration of the process efficiency was studied in the concentration range of 0.01–0.1 M (2 mL of peroxide solution was used). Figure 11a shows the kinetic curves of the discoloration process, whereas Fig. 11b, the removal efficiency of the process at different H_2_O_2_ doses at pH = 3.

The discoloration experiments presented in Fig. 11 were performed in the most concentrated dye solution studied in this work (0.5 M). Compared with the data in Fig. 11, where the maximum removal efficiency was slightly higher than 20% with the same amount of adsorbent (0.010 g), in presence of peroxide almost complete discoloration was achieved (94%). The substantial enhancement of the removal efficiency is not a result only of the changed pH in this case (pH = 3), but as well because, of the dye degradation that occurred by the OH radicals. Figure 11a shows that the rate of the discoloration process increased with the addition of peroxide and with increasing its concentration. Accordingly, the removal efficiency increased, too (Fig. 11b). This can be attributed to the increased amount of hydroxyl radicals. However, with a further increase in H_2_O_2_ concentration from 0.05 to 0.1 M, the dye removal efficiency did not change. In excess quantity of H_2_O_2_, it acted as a scavenger of ^•^OH via reacting with ^•^OH to produce ^•^OOH^55,56^. The efficiency of the hybrid catalyst was compared with neat Fe_3_O_4_ nanoparticles using H_2_O_2_ concentration of 0.5 M. Initially, the neat magnetic nanoparticles presented improved activity, which after 5 min became saturated and no further AG removal was observed. The improved initial activity may be a result on the much higher concentration of neat nanoparticles (we added the same quantity of nanoparticle as the hybrid catalysts, in which at least half is inactive rGO substrate).

**Figure 12:**
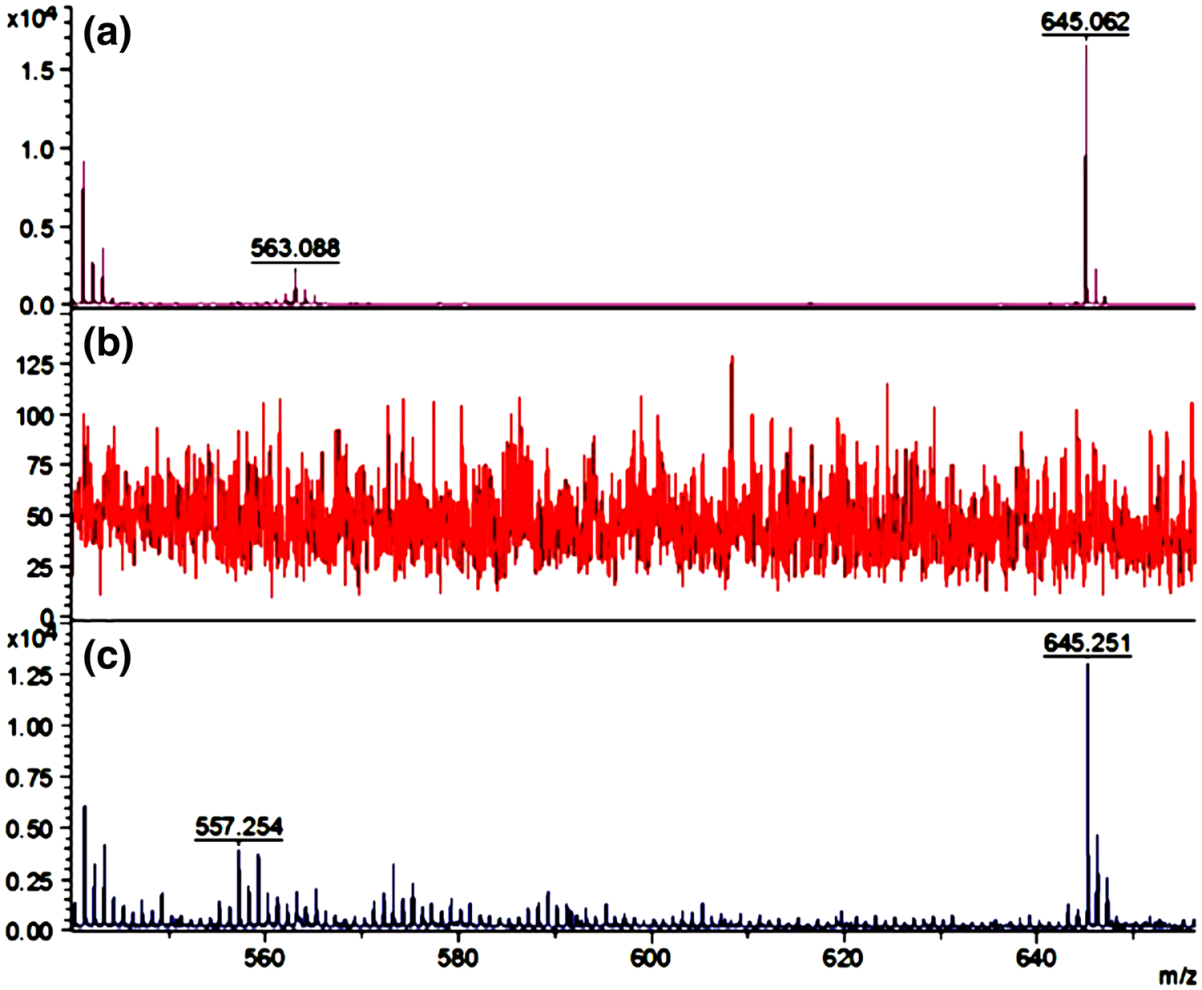
Comparison of average mass spectra of (**a**) AG-25 (**b**) aqueous AG-25 solution after 3 h of Fenton’s reaction over Ch-rGO/Fe_3_O_4_ in the presence of 0.1 M H_2_O_2_ (**c**) AG-25 after 3 h with H_2_O_2_ (0.1 M) and 3D-rGO (0.010 g).

The aqueous solution after 3 h of discoloration/degradation process was subjected to MALDI–TOF–MS analysis to check if the degradation of AG-25 proceeds and to identify the degradation products. This process was compared with the discoloration/degradation performed by neat 3D-rGO nanostructure in presence of the peroxide at the same concentration (0.1 M H_2_O_2_). AG-25 original dye was analyzed by MALDI–TOF–MS, too. The mass spectra obtained are presented in Fig. 12. It is worth mentioning that MALDI–TOF–MS is especially useful for detecting degradation products of AG-25, due to a lack of fragmentation of the molecules during analysis.

In Fig. 12a, where the mass spectrum of AG-25 dye is shown, the characteristic peak at m/z 645 (AG-25 [M + Na] specie) can be observed. Mass spectra of AG-25 solution after 3 h of Fenton’s reaction over hybrid catalysts Ch-rGO/Fe_3_O_4_ in the presence of 0.1 M H_2_O_2_ is shown in Fig. 12b. The lack of characteristic peaks of AG-25 demonstrates that it was completely degraded. Additionally, no peaks of secondary products were observed. On contrary, in Fig. 12c, where AG-25 aqueous solution after 3 h discoloration by the neat 3D-rGO in presence of H_2_O_2_ is shown, the AG-25 characteristic peak at m/z 645 can be observed, indicating that 3D-rGO/H_2_O_2_ did not completely remove the dye from the solution. Moreover, in Fig. 12c, numerous new peaks arising from the dye degradation appeared, pointing out that the dye was only partially degraded by the peroxide.

**Figure 13:**
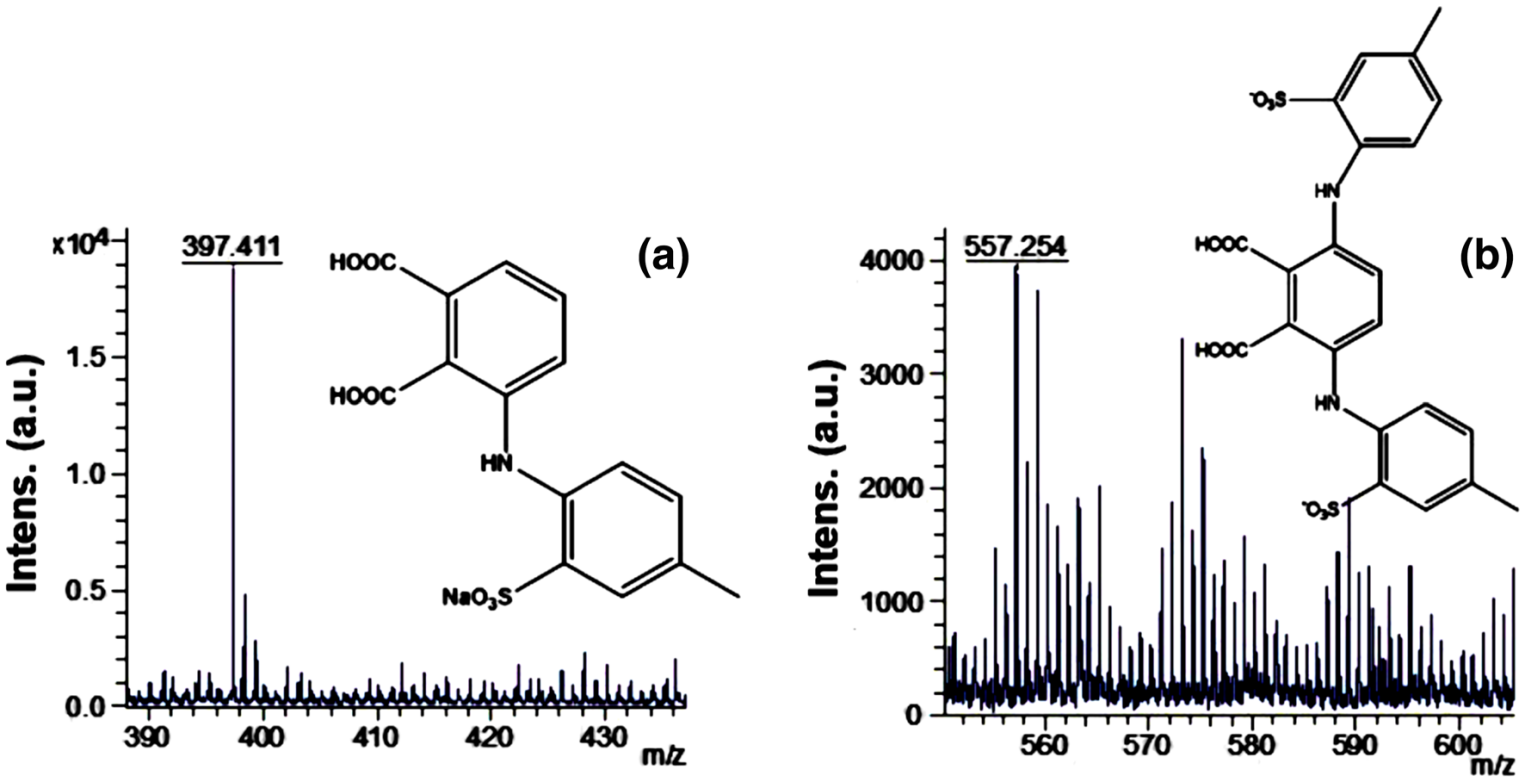
Mass spectra and the assignment of peaks observed in aqueous solution after discoloration/degradation of AG-25 by H_2_O_2_ in the presence of 3D-rGO.

The mass spectra of the aqueous solution treated with neat 3D-rGO/H_2_O_2_ were further analysed to check the type of the products formed. Figure 13 presents the zoomed area of some characteristic peaks (molar mass of 397 and 557 m/z) and their assignments. Likely, the observed AG-25 degradation products contain multiple aromatic rings. These intermediate products behave as competitors to AG-25 for adsorption sites, and react with the hydroxyl radicals, acting as hydroxyl radical scavengers, decreasing the removal efficiency^55,56^.

**Figure 14:**
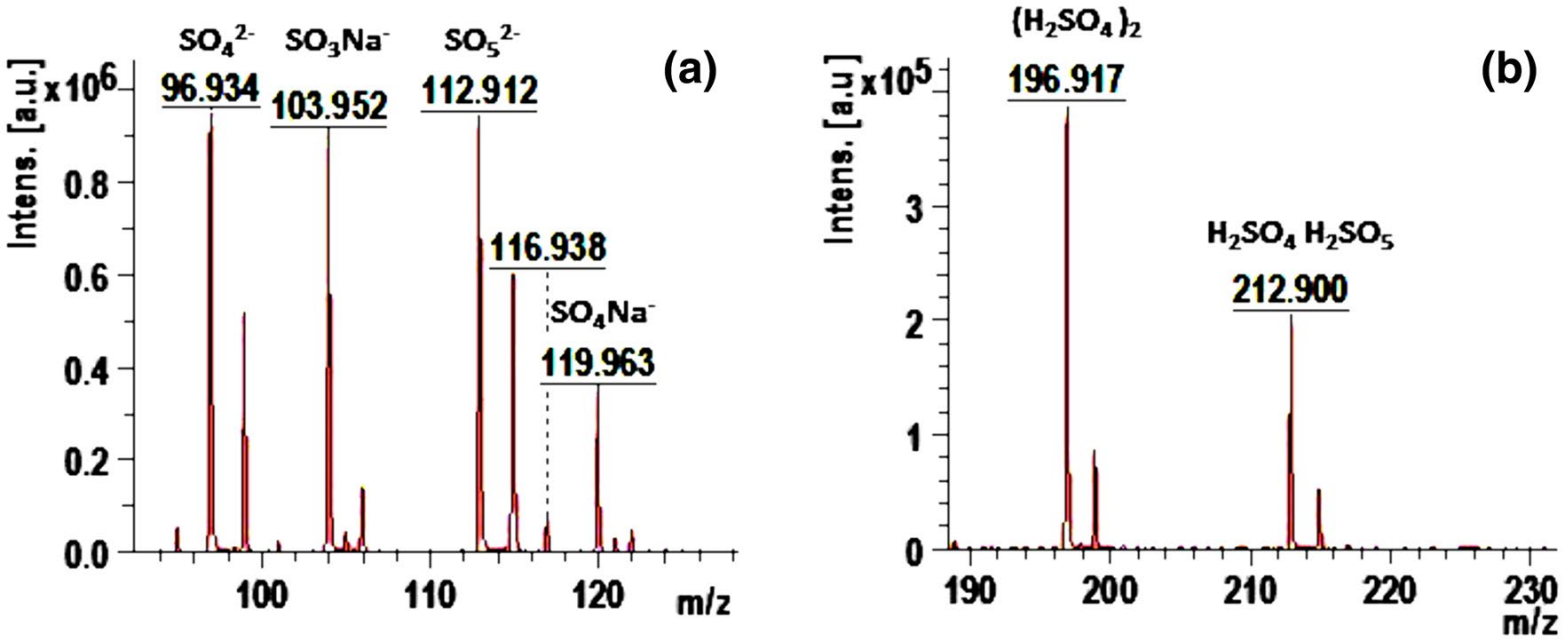
Zoomed areas of the MALDI–TOF–MS spectra of aqueous solution after 3 h discoloration/degradation of AG-25 dye solution at pH = 3, over Ch-rGO/Fe_3_O_4_ nanocatalysts (0.010 g) and H_2_O_2_ (0.1 M).

On the other hand, AG-25 was degraded completely by the Ch-rGO/Fe_3_O_4_–H_2_O_2_. To check the type of degradation products, in Fig. 14, the zoomed area of the peaks that appeared in Fig. 14b are shown. The observed products are NaSO_3_^−^, SO_4_^2−^, SO_5_^2−^, NaSO_4_^−^ (Fig. 14a). In addition, sulphuric acid clusters were identified, which could be formed from degradation products interactions (Fig. 14b). No larger organic degradation product with aromatic rings or conjugated aromatic rings was detected after Fenton’s reaction, suggesting that not only a full AG-25 degradation, but as well degradation of all intermediate products. In other words, Ch-rGO/Fe_3_O_4_ ensures complete mineralization of AG-25 in less than 30 min in as concentrated aqueous solution as 0.5 M, with only 0.010 g composite Fenton catalyst and in the presence of 0.1 M H_2_O_2_. Moreover, this process was performed at room temperature without any additional energy source.

In the previous study, the excellent Fenton catalyst activity of the composite based on rGO and Fe_3_O_4_ nanoparticles was explained on the base of the charge transfer complex established between both components, which facilitate the electron transfer and regeneration of ferrous ions after ^•^OH creation^35^. The maximum efficiency was achieved for 0.12 mg mL^−1^ concentration of acid red dye, ensuring complete discoloration and partial degradation. In this study, the catalytic activity was further enhanced by the promotion of interactions between the 3D aerogel structure and the organic dye that allowed efficient discoloration and complete mineralization of 0.5 M AG-25 dye solution, even in the more concentrated dye solution and more chemically complex dye.

Concerning AG degradation by Fenton reaction over catalysts based on magnetic nanoparticles, few relatively recent reports were found. In a recent article^57^, the authors reported a green synthesis of magnetic nanoparticles Fenton catalyst from neem leaf extract. However, the synthesis is based on the use of solvents (versus water-based procedures in this work) and the procedure seems costly. Under similar conditions, this catalyst removed the AG-25 dye in 100 min (vs 30 min in our work). The authors in this work improved the efficiency of the process by applying sonication energy and achieved degradation in 20 min, however by GC–MS multiple aromatic secondary products were identified (versus complete mineralization achieved in our study without use of additional energy, UV light or sonication). The use of hybrid catalysts was reported for Fenton AG degradation, either magnetic nanoparticles were inserted in alumina gel ^58^, or clays^59^. In all cases, the synthesis procedure is both time and energy-consuming. The authors reported high or complete AG mineralization based on TOC measurement. Nevertheless, it does not exclude the existence of secondary products that were not studied.

**Figure 15:**
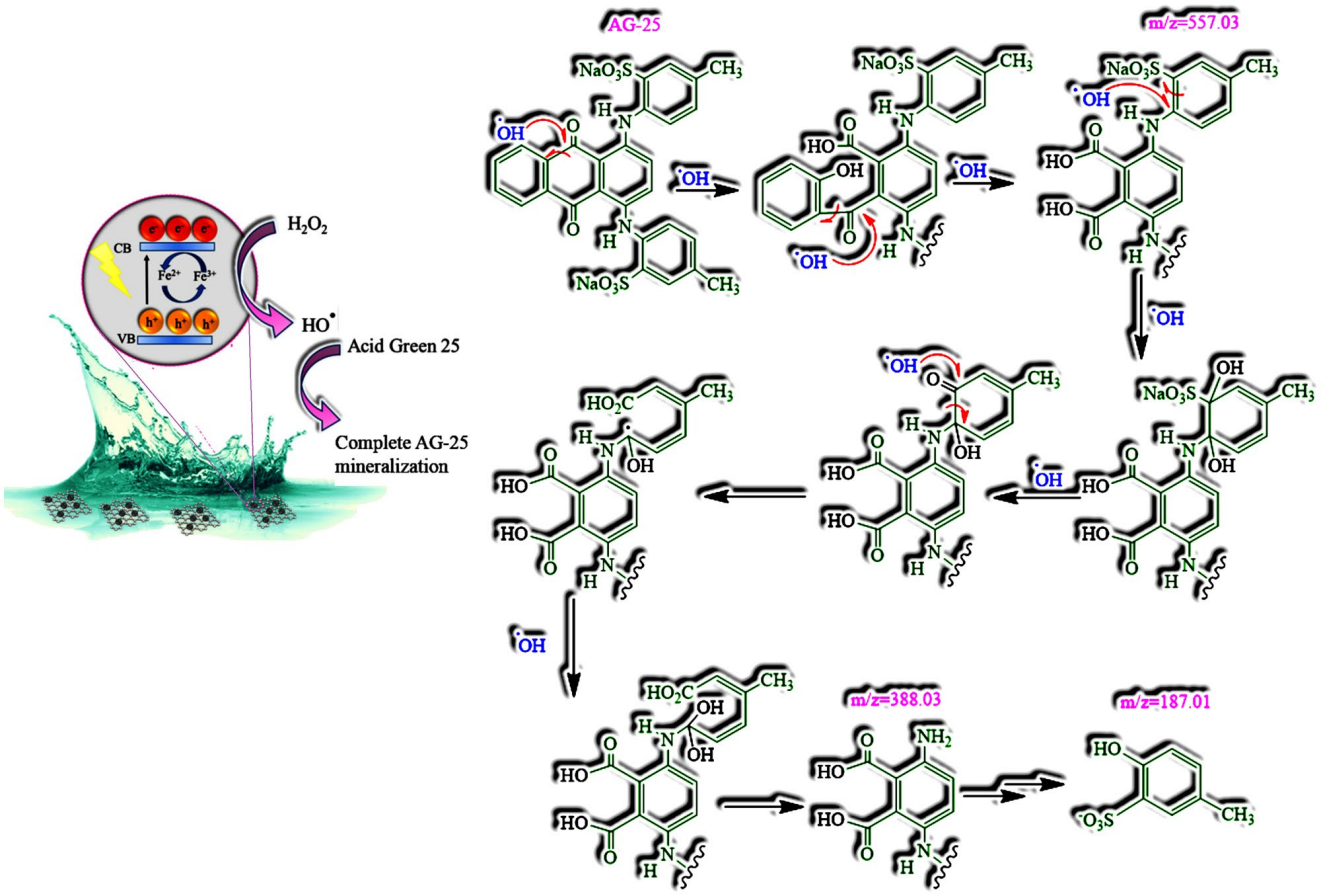
Proposed mechanism of AG-25 degradation in the 3D rGO/Fe_3_O_4_/H_2_O_2_ system.

The proposed AG-25 degradation mechanism, based on rGO–Fe_3_O_4_ charge transfer complex is shown in Fig. 15. The efficiency of the charge transfer was improved due to the presence of the C–O–Fe bond, the formation of which is promoted by the production method of the in situ creation of the Fe_3_O_4_ NP.

In Fig. 15, the detailed mechanism of AG-25 degradation is shown for the first time, up to the best knowledge of the authors. The degradation mechanism of AG-25 presented in literature is based on the formation of OH radicals by Fenton reaction that induces Ag-25 mineralization, without analysis of the degradation routes and secondary products^60,61^. Degradation paths of similar molecules, such as Acid Green 1^62^ or Malachite Green^63^, were proposed. Discoloration of Acid Green 1 by atmospheric oxidation was proposed to occur through direct oxidation of amino and sulphonic acid moieties to hydroquinones and quinones. The C=N group in Acid Green 1 was proposed to undergo oxidative hydrolysis with H_2_O_2_ and form corresponding phenolic compound and finally quinones, whereas, sulfonic acid –(C–SO_2_–OH–) or it sodium salt present in the dye molecule undergo oxidative hydrolysis to corresponding hydroxyl and carbonyl compounds. Alternatively, the degradation mechanism reported for Malachite Green dye was based on three main reaction types: decomposition of conjugated structure, accompanying with minor N-demethylation reactions and adduction reactions.

Based on the main secondary products identified at m/z 557.03, 388.08 and 187.01 (Fig. 14) by MALDI–TOF–MS, we proposed a detailed AG-25 degradation mechanism. It was accomplished throughout OH radicals attack to the intermediate cyclic structure in the conjugated moiety of AG-25. Consequently, phenol and carboxylic acid containing moieties were created. At high OH radical concentration, this degradation continues until a molecule with a smaller structure is formed. The sodium salt of sulfonic acid (C–SO_2_–O–Na+) moiety undergoes oxidative hydrolysis to corresponding hydroxyl and carbonyl compounds.

## Conclusions

In this study, 3D composite structures made of rGO and magnetic nanoparticles Fe_3_O_4_ were synthesized by a self-assembly process, during the simultaneous reduction of GO nanoplatelets and precursor for magnetic nanoparticles. The resulting 3D materials presented the porous structure and high surface area, which were affected by the synthesis conditions and the reduction process type (either thermal or combined chemical and thermal reduction process).

The selected nanocomposite was applied as adsorbent of azo dye AG-25 from model aqueous solution, and the adsorption process was optimized using a selection of the quantity of adsorbent and pH of the dye aqueous solution. It was shown that, when pH is in-between the pKa of carboxylic groups on graphene and pKa of the sulfonic acid group in AG-25, the removal efficiency is maximized. In such conditions, ion–dipole interactions are promoted. The proposed adsorption mechanism is based on the mentioned electrostatic interactions and the possibility of π–π bonding between the neat graphenic areas in rGO and the conjugated aromatic structures of AG-25.

In the next step, the 3D composite rGO/Fe_3_O_4_ was applied as a Fenton catalyst in the presence of H_2_O_2_ for the AG-25 degradation and the performance was compared with the degradation of the dye by H_2_O_2_ in presence of a neat 3D-rGO structure. By MALDI–TOF–MS analysis, it was determined that complete mineralization of AG-25 and all intermediate products were attained by the Fenton reaction by rGO/Fe_3_O_4_/H_2_O_2_ in as concentrated AG-25 solution as 0.5 mg mL^−1^, whereas only partial discoloration and presence of multiple reaction intermediates were observed in the solution after rGO/H_2_O_2_ reaction. The complete degradation was achieved in less than 30 min, at room temperature, without any external energy added in the system. The excellent performance was assigned to the rGO-magnetic nanoparticles charge transfer complex, built up by the establishment of the C–O–Fe covalent bond, promoted further by selecting optimal discoloration/degradation conditions. Finally, an oxidative degradation mechanism for AG-25 was proposed, based on the identified secondary products by MALDI–TOF–MS.

## Supplementary Information

Supplementary Figures.

## References

[1] Wrolstad, R. E. Color and pigment analyses in fruit products. (1993).

[2] Maldonado-Larios L (2020). Electrochemically-assisted fabrication of titanium-dioxide/polyaniline nanocomposite films for the electroremediation of congo red in aqueous effluents. Synth. Met..

[3] Song W (2016). Adsorption–desorption behavior of magnetic amine/Fe_3_O_4_ functionalized biopolymer resin towards anionic dyes from wastewater. Biores. Technol..

[4] Mukherjee A, Adak MK, Dhak P, Dhak D (2020). A simple chemical method for the synthesis of Cu^2+^ engrafted MgAl2O4 nanoparticles: Efficient fluoride adsorbents, photocatalyst and latent fingerprint detection. J. Environ. Sci..

[5] Pathania D, Sharma S, Singh P (2017). Removal of methylene blue by adsorption onto activated carbon developed from Ficus carica bast. Arab. J. Chem..

[6] Gupta VK, Kumar R, Nayak A, Saleh TA, Barakat M (2013). Adsorptive removal of dyes from aqueous solution onto carbon nanotubes: A review. Adv. Colloid Interface Sci..

[7] Peng N (2016). Superabsorbent cellulose–clay nanocomposite hydrogels for highly efficient removal of dye in water. ACS Sustain. Chem. Eng..

[8] Hsu T-C (2008). Adsorption of an acid dye onto coal fly ash. Fuel.

[9] Murray A, Örmeci B (2018). Competitive effects of humic acid and wastewater on adsorption of Methylene Blue dye by activated carbon and non-imprinted polymers. J. Environ. Sci..

[10] Tara N, Siddiqui SI, Rathi G, Chaudhry SA, Asiri AM (2020). Nano-engineered adsorbent for the removal of dyes from water: A review. Curr. Anal. Chem..

[11] Homaeigohar S (2020). The nanosized dye adsorbents for water treatment. Nanomaterials.

[12] Zare K (2015). A comparative study on the basis of adsorption capacity between CNTs and activated carbon as adsorbents for removal of noxious synthetic dyes: A review. J. Nanostruct. Chem..

[13] Allen MJ, Tung VC, Kaner RB (2010). Honeycomb carbon: A review of graphene. Chem. Rev..

[14] Chowdhury S, Balasubramanian R (2014). Recent advances in the use of graphene-family nanoadsorbents for removal of toxic pollutants from wastewater. Adv. Colloid Interface Sci..

[15] Lai KC, Lee LY, Hiew BYZ, Thangalazhy-Gopakumar S, Gan S (2019). Environmental application of three-dimensional graphene materials as adsorbents for dyes and heavy metals: Review on ice-templating method and adsorption mechanisms. J. Environ. Sci..

[16] Mahmoudi E (2020). Simultaneous removal of Congo red and cadmium (II) from aqueous solutions using graphene oxide–silica composite as a multifunctional adsorbent. J. Environ. Sci..

[17] Kyzas GZ, Deliyanni EA, Bikiaris DN, Mitropoulos AC (2018). Graphene composites as dye adsorbents. Chem. Eng. Res. Des..

[18] Ramesha G, Kumara AV, Muralidhara H, Sampath S (2011). Graphene and graphene oxide as effective adsorbents toward anionic and cationic dyes. J. Colloid Interface Sci..

[19] Robati D (2016). Removal of hazardous dyes-BR 12 and methyl orange using graphene oxide as an adsorbent from aqueous phase. Chem. Eng. J..

[20] Gulati A, Kakkar R (2020). Graphene-based adsorbents for water remediation by removal of organic pollutants: Theoretical and experimental insights. Chem. Eng. Res. Des..

[21] Su S (2019). A novel graphene oxide-carbon nanotubes anchored α-FeOOH hybrid activated persulfate system for enhanced degradation of Orange II. J. Environ. Sci..

[22] Hong PN, Minh DN, Van Hung N, Minh PN, Khoi PH (2017). Carbon nanotube and graphene aerogels—The world’s 3D lightest materials for environment applications: A review. Int. J. Mater. Sci. Appl..

[23] Ye S, Liu Y, Feng J (2017). Low-density, mechanical compressible, water-induced self-recoverable graphene aerogels for water treatment. ACS Appl. Mater. Interfaces.

[24] Dong S (2018). Controlled synthesis of flexible graphene aerogels macroscopic monolith as versatile agents for wastewater treatment. Appl. Surf. Sci..

[25] Jiang L, Wen Y, Zhu Z, Liu X, Shao W (2020). A Double cross-linked strategy to construct graphene aerogels with highly efficient methylene blue adsorption performance. Chemosphere.

[26] Li Z-J (2017). Enhanced photocatalytic removal of uranium (VI) from aqueous solution by magnetic TiO_2_/Fe_3_O_4_ and its graphene composite. Environ. Sci. Technol..

[27] Pourjavadi A, Nazari M, Hosseini SH (2015). Synthesis of magnetic graphene oxide-containing nanocomposite hydrogels for adsorption of crystal violet from aqueous solution. RSC Adv..

[28] Li S (2015). Highly efficient degradation of organic dyes by palladium nanoparticles decorated on 2D magnetic reduced graphene oxide nanosheets. Dalton Trans..

[29] He K (2018). Three-dimensional graphene supported catalysts for organic dyes degradation. Appl. Catal. B.

[30] Shan D (2018). Hydrophilic and strengthened 3D reduced graphene oxide/nano-Fe_3_O_4_ hybrid hydrogel for enhanced adsorption and catalytic oxidation of typical pharmaceuticals. Environ. Sci. Nano.

[31] Lu J, Zhou Y, Lei J, Ao Z, Zhou Y (2020). Fe_3_O_4_/graphene aerogels: A stable and efficient persulfate activator for the rapid degradation of malachite green. Chemosphere.

[32] Zhang F, Xue X, Huang X, Yang H (2020). Adsorption and heterogeneous Fenton catalytic performance for magnetic Fe_3_O_4_/reduced graphene oxide aerogel. J. Mater. Sci..

[33] Huyen LT, Duc DS, Hoan NX, Tho NH, Viet NX (2019). Synthesis of Fe_3_O_4_-reduced graphene oxide modified tissue-paper and application in the treatment of methylene blue. VNU J. Sci. Nat. Sci. Technol..

[34] Li Y, Zhang R, Tian X, Yang C, Zhou Z (2016). Facile synthesis of Fe_3_O_4_ nanoparticles decorated on 3D graphene aerogels as broad-spectrum sorbents for water treatment. Appl. Surf. Sci..

[35] Sadegh F (2020). A green synthesis of nanocatalysts based on reduced graphene oxide/magnetic nanoparticles for the degradation of Acid Red 1. RSC Adv..

[36] Zhang M-H, Dong H, Zhao L, Wang D-X, Meng D (2019). A review on Fenton process for organic wastewater treatment based on optimization perspective. Sci. Total Environ..

[37] Rezaei F, Vione D (2018). Effect of pH on zero valent iron performance in heterogeneous fenton and fenton-like processes: A review. Molecules.

[38] Szpyrkowicz L, Juzzolino C, Kaul SN (2001). A comparative study on oxidation of disperse dyes by electrochemical process, ozone, hypochlorite and Fenton reagent. Water Res..

[39] Lu H-F, Chen H-F, Kao C-L, Chao I, Chen H-Y (2018). A computational study of the Fenton reaction in different pH ranges. Phys. Chem. Chem. Phys..

[40] Barrera-Díaz C, Canizares P, Fernández F, Natividad R, Rodrigo MA (2014). Electrochemical advanced oxidation processes: an overview of the current applications to actual industrial effluents. J. Mex. Chem. Soc..

[41] Jung YS, Lim WT, Park JY, Kim YH (2009). Effect of pH on Fenton and Fenton-like oxidation. Environ. Technol..

[42] Vasquez-Medrano R, Prato-Garcia D, Vedrenne M (2018). Advanced Oxidation Processes for Waste Water Treatment.

[43] Thomas N, Dionysiou DD, Pillai SC (2020). Heterogeneous Fenton catalysts: A review of recent advances. J. Hazard. Mater..

[44] Nworie F, Nwabue F, Oti W, Mbam E, Nwali B (2019). Removal of methylene blue from aqueous solution using activated rice husk biochar: Adsorption isotherms, kinetics and error analysis. J. Chil. Chem. Soc..

[45] Politakos N (2020). Graphene-based monolithic nanostructures for CO_2_ capture. Ind. Eng. Chem. Res..

[46] Cychosz KA, Thommes M (2018). Progress in the physisorption characterization of nanoporous gas storage materials. Engineering.

[47] Grosman A, Ortega C (2005). Nature of capillary condensation and evaporation processes in ordered porous materials. Langmuir.

[48] Zubir NA, Yacou C, Motuzas J, Zhang X, Da Costa JCD (2014). Structural and functional investigation of graphene oxide–Fe_3_O_4_ nanocomposites for the heterogeneous Fenton-like reaction. Sci. Rep..

[49] Kumar R, Singh RK, Vaz AR, Savu R, Moshkalev SA (2017). Self-assembled and one-step synthesis of interconnected 3D network of Fe_3_O_4_/reduced graphene oxide nanosheets hybrid for high-performance supercapacitor electrode. ACS Appl. Mater. Interfaces.

[50] Cheng G (2012). The GO/rGO–Fe_3_O_4_ composites with good water-dispersibility and fast magnetic response for effective immobilization and enrichment of biomolecules. J. Mater. Chem..

[51] Liu L (2018). An electrochemical sensor for diphenylamine detection based on reduced graphene oxide/Fe_3_O_4_-molecularly imprinted polymer with 1, 4-Butanediyl-3, 3′-bis-l-vinylimidazolium dihexafluorophosphate ionic liquid as cross-linker. Polymers.

[52] Namazian M, Halvani S (2006). Calculations of pKa values of carboxylic acids in aqueous solution using density functional theory. J. Chem. Thermodyn..

[53] Rayne S, Forest K, Friesen K (2009). Extending the semi-empirical PM6 method for carbon oxyacid pKa prediction to sulfonic acids: Application towards congener-specific estimates for the environmentally and toxicologically relevant C1 through C8 perfluoroalkyl derivatives. Nat. Prec..

[54] Qiu B, Li Q, Shen B, Xing M, Zhang J (2016). Stöber-like method to synthesize ultradispersed Fe_3_O_4_ nanoparticles on graphene with excellent Photo-Fenton reaction and high-performance lithium storage. Appl. Catal. B.

[55] Yang X (2015). Rapid degradation of methylene blue in a novel heterogeneous Fe_3_O_4_@ rGO@ TiO_2_-catalyzed photo-Fenton system. Sci. Rep..

[56] Qin Y, Long M, Tan B, Zhou B (2014). RhB adsorption performance of magnetic adsorbent Fe_3_O_4_/RGO composite and its regeneration through a fenton-like reaction. Nano-Micro Lett..

[57] Prakash LV (2021). Ultrasound aided heterogeneous Fenton degradation of Acid Blue 15 over green synthesized magnetite nanoparticles. Sep. Purif. Technol..

[58] Ramos Preza C (2014). Mineralization of Acid Green 50 by Fe_2_O_3_–Al_2_O_3_ as a highly active hetero-Fenton catalyst. Desalin. Water Treat..

[59] Azmi N, Ayodele O, Vadivelu V, Asif M, Hameed B (2014). Fe-modified local clay as effective and reusable heterogeneous photo-Fenton catalyst for the decolorization of Acid Green 25. J. Taiwan Inst. Chem. Eng..

[60] Bhatti HN, Iram Z, Iqbal M, Nisar J, Khan MI (2020). Facile synthesis of zero valent iron and photocatalytic application for the degradation of dyes. Mater. Res. Express.

[61] Chen F, Ma W, He J, Zhao J (2002). Fenton degradation of malachite green catalyzed by aromatic additives. J. Phys. Chem. A.

[62] Thasilu K, Karthikeyan J (2016). Decolorisation and degradation of C.I. acid green 1 by H_2_O_2_ and Fenton oxidation processes. Am. J. Environ. Eng..

[63] Ming Y, Gui JS, Chao YY, Cheng D, Gang SCh, Zhong G, Chao H, Huan Q, Xu HB (2009). Microwave-enhanced H_2_O_2_-based process for treating aqueous malachite greensolutions: Intermediates and degradation mechanism. J. Hazard. Mater..

