# Supplementary material for: Toward enhanced catalytic activity of magnetic nanoparticles integrated into 3D reduced graphene oxide for heterogeneous Fenton organic dye degradation
Source: Sci Rep. 2021 Sep 15;11:18343. doi: 10.1038/s41598-021-97712-7 (PMC8443561; doi:10.1038/s41598-021-97712-7)
Supplement: Supplementary file 1 — Supplementary Figures. [file 41598_2021_97712_MOESM1_ESM.docx]

**Supporting information**

**Towards enhanced catalytic activity of magnetic nanoparticles integrated into 3D reduced graphene oxide for heterogeneous Fenton organic dye degradation**

*Fatemeh Sadegh,^a,b^ Nikolaos Politakos,^a^* *Estibaliz Gonzalez de San Roman,^a^ Oihane Sanz,^c^ Ali Reza Modarresi-Alam,^b,d^ and Radmila Tomovska^a,e,*^*

^a^ POLYMAT, Facultad de Ciencias, Químicas, University of the Basque Country UPV/EHU, Joxe Mari Korta, Center - Avda. Tolosa, 72, San Sebastian, 20018, Spain. E-mail: [radmila.tomovska@ehu.es](mailto:radmila.tomovska@ehu.es)

^b^ Organic and Polymer Research Laboratory, Department of Chemistry, Faculty of Science, University of Sistan and Baluchestan, Zahedan, Iran.

^c^ Departamento de Química Aplicada, Facultad de Ciencias, Químicas, University of the Basque Country, UPV/EHU, P. Manuel de Lardizabal 3, San Sebastian, 20018, Spain.

^d^ Renewable Energies Research Institute, University of Sistan and Baluchestan, Zahedan, Iran.

^e^ Ikerbasque, Basque Foundation for Science, Maria Diaz de Haro 3, Bilbao, 48013, Spain.


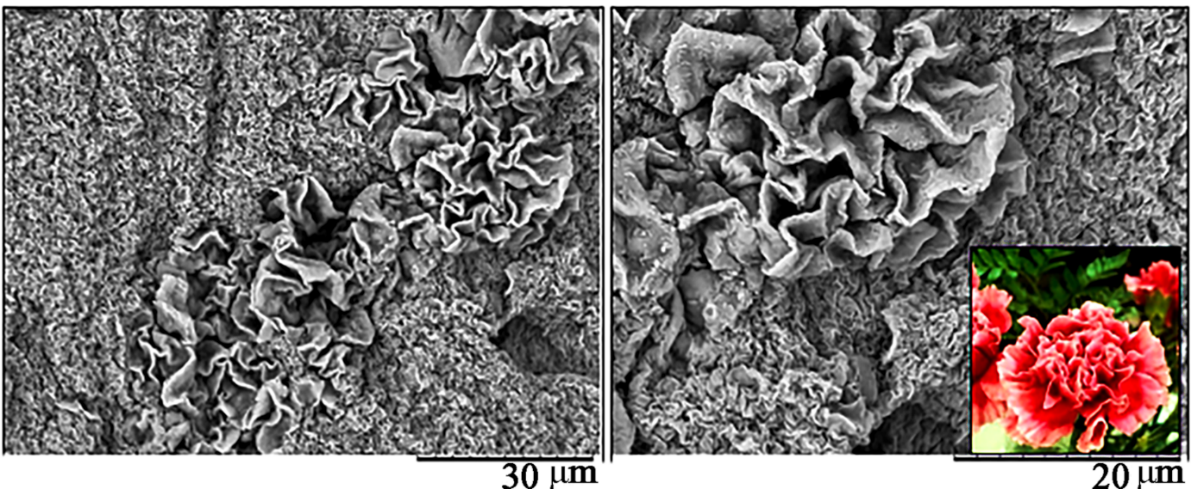


**Figure S1.** SEM micrographs of Ch-rGO/Fe_3_O_4_ nanostructure obtained by heat-drying under lower (left) and higher (right) magnification. The inset in the right image show a photo of flower with similar structuring.


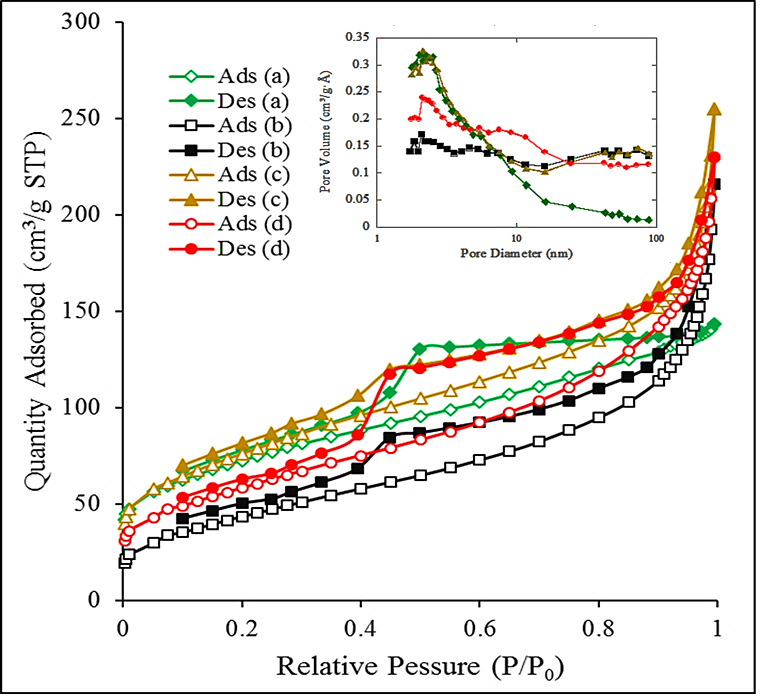


**Figure S2**. Adsorption-desorption isotherm of N_2_ (inset shows pore size distribution) of (**a**) neat 3D- rGO; (**b**) Th-rGO/Fe_3_O_4_/PVP; (**c**) Ch-rGO/Fe_3_O_4_; and (**d**) Th-rGO/Fe_3_O_4_. In the inset, pore size distributions of the same materials are presented.


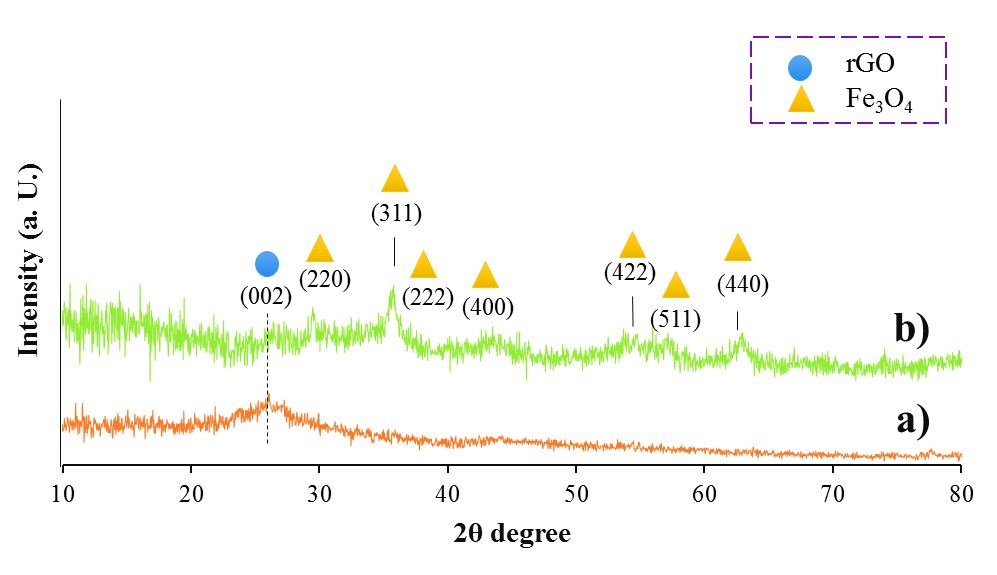


**Figure S3**. XRD patterns of (**a**) neat rGO and (**b**) Ch-rGO/Fe_3_O_4_.


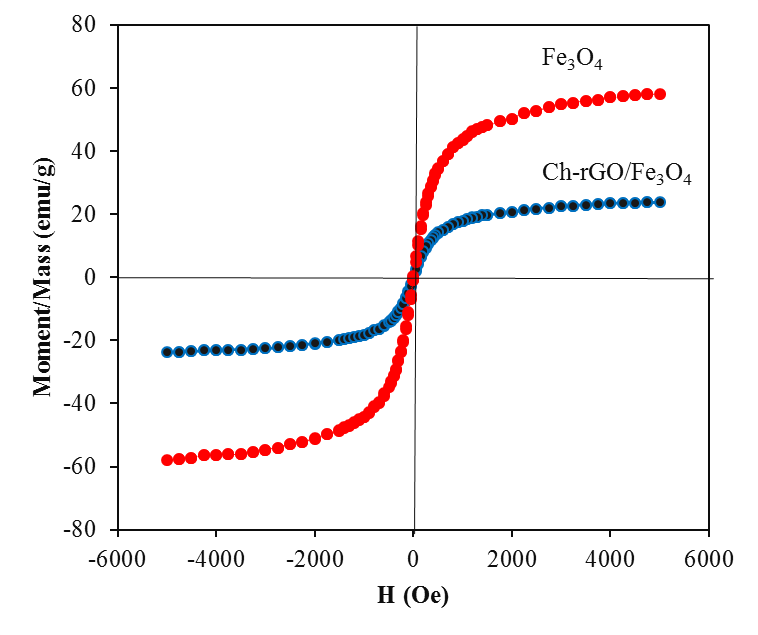


**Figure S4.** Magnetization curves of Fe_3_O_4_ and Ch-RGO/Fe_3_O_4_ at room temperature.


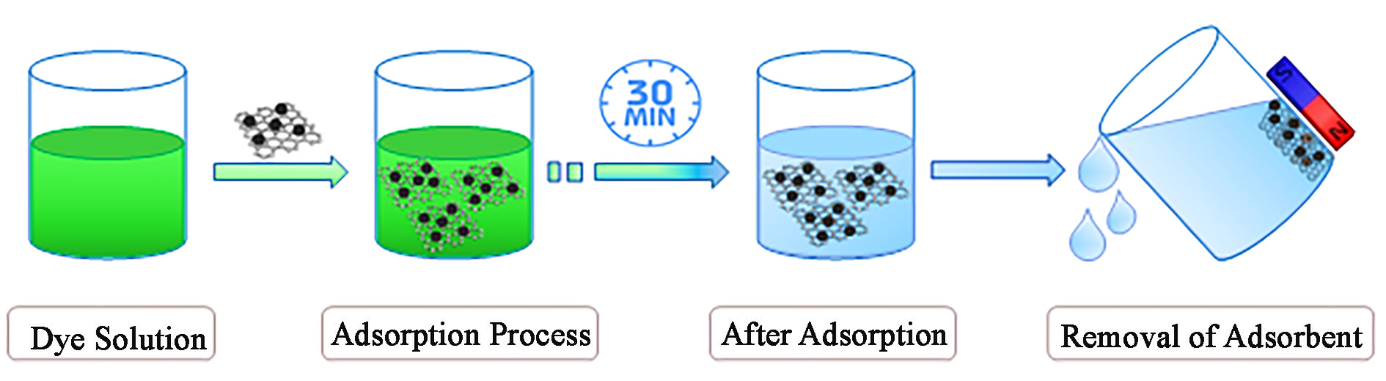
 **Figure S5**. Schematic illustration of the adsorption process for the AG-25 using Ch-rGO/Fe_3_O_4_.


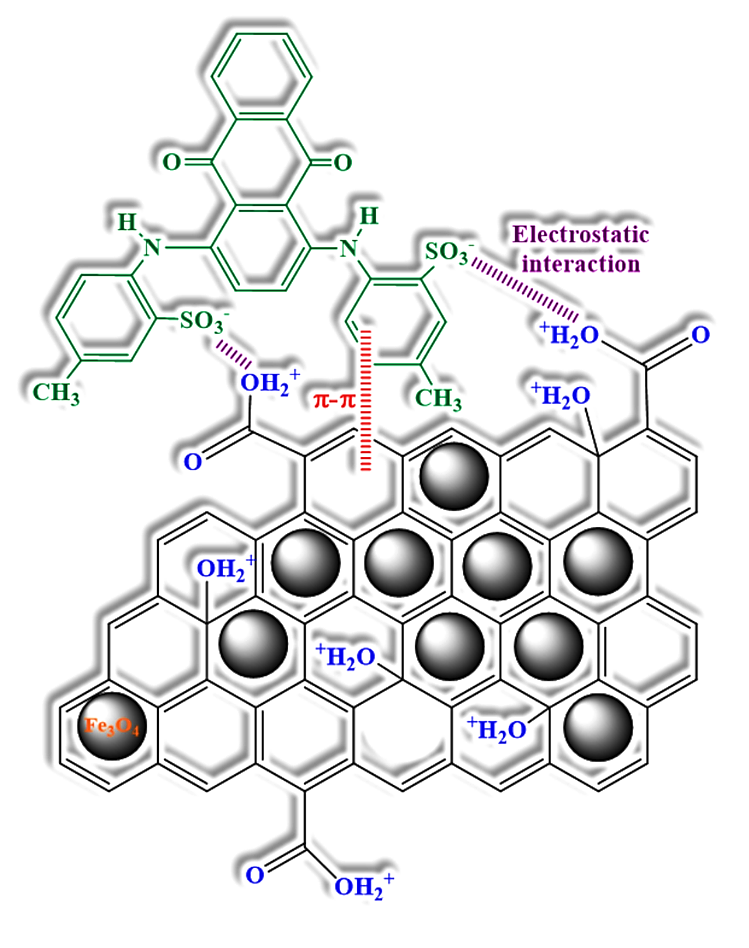


**Figure S6**. Adsorption mechanism of the AG-25 by Ch-rGO/Fe_3_O_4_ nanostructure.
